# Autocrine phosphatase PDP2 inhibits ferroptosis by dephosphorylating ACSL4 in the Luminal A Breast Cancer

**DOI:** 10.1371/journal.pone.0299571

**Published:** 2024-03-11

**Authors:** Jun-Jie Zhu, Feng-Ying Huang, Hengyu Chen, Yun-long Zhang, Ming-Hui Chen, Ri-Hong Wu, Shu-Zhen Dai, Gui-Sheng He, Guang-Hong Tan, Wu-Ping Zheng

**Affiliations:** Department of Breast and Thyroid Surgery, The Second Affiliated Hospital and Key Laborato1y of Tropical Translational Medicine of Ministry of Education & School of Tropical Medicine, Hainan Medical University, Haikou, China; Middle East Technical University, TURKEY

## Abstract

Phosphatases can dephosphorylate phosphorylated kinases, leading to their inactivation, and ferroptosis is a type of cell death. Therefore, our aim is to identify phosphatases associated with ferroptosis by analyzing the differentially expressed genes (DEGs) of the Luminal A Breast Cancer (LumABC) cohort from the Cancer Genome Atlas (TCGA). An analysis of 260 phosphatase genes from the GeneCard database revealed that out of the 28 DEGs with high expression, only the expression of pyruvate dehydrogenase phosphatase 2 (PDP2) had a significant correlation with patient survival. In addition, an analysis of DEGs using gene ontology, Kyoto Encyclopedia of Genes and Genomes and gene set enrichment analysis revealed a significant variation in the expression of ferroptosis-related genes. To further investigate this, we analyzed 34 ferroptosis-related genes from the TCGA-LumABC cohort. The expression of long-chain acyl-CoA synthetase 4 (ACSL4) was found to have the highest correlation with the expression of PDP2, and its expression was also inversely proportional to the survival rate of patients. Western blot experiments using the MCF-7 cell line showed that the phosphorylation level of ACSL4 was significantly lower in cells transfected with the HA-PDP2 plasmid, and ferroptosis was correspondingly reduced (p < 0.001), as indicated by data from flow cytometry detection of membrane-permeability cell death stained with 7-aminoactinomycin, lipid peroxidation, and Fe^2+^. Immunoprecipitation experiments further revealed that the phosphorylation level of ACSL4 was only significantly reduced in cells where PDP2 and ACSL4 co-precipitated. These findings suggest that PDP2 may act as a phosphatase to dephosphorylate and inhibit the activity of ACSL4, which had been phosphorylated and activated in LumABC cells. Further experiments are needed to confirm the molecular mechanism of PDP2 inhibiting ferroptosis.

## Introduction

Breast cancer is now the most commonly diagnosed cancer worldwide, accounting for 11.7% of all cancer cases [1]. This is largely due to changes in the living environment and the increasing proportion of women in industrial production, which have led to delayed childbirth and declining birth rates. Luminal A breast cancer (LumABC) is a molecular subtype of breast cancer that is distinct from other subtypes, characterized by high expression of estrogen receptor (ER) and/or progesterone receptor (PR), low expression of human epidermal growth factor receptor 2 (HER2), and a low proliferation index, such as Ki-67 [2]. It is generally associated with a better prognosis than other breast cancer subtypes, such as Luminal B, HER2-positive, and triple-negative, with lower recurrence rates and improved survival [3–5]. Furthermore, LumABC is more likely to respond to endocrine therapies [6]. Despite this, research on this subtype of breast cancer has been relatively neglected, so further investigation is necessary.

Metabolism in cancerous cells is distinct from that of normal cells, as they primarily generate energy through glycolysis instead of oxidative phosphorylation in the mitochondria—a phenomenon known as the Warburg effect [7–9]. This is due to the inhibition of the pyruvate dehydrogenase complex (PDC) by pyruvate dehydrogenase kinase (PDK). Pyruvate dehydrogenase (PDH) is the key enzyme of the PDC, which catalyzes the decarboxylation of pyruvate to form acetyl-CoA, which then enters the tricarboxylic acid cycle to produce ATP [10]. Pyruvate Dehydrogenase Phosphatase (PDP) has been found to activate or enhance the activity of PDH, allowing glycolysis to enter the tricarboxylic acid cycle and generate more ATP [10–12]. However, the involvement of PDP in the growth and metastasis of breast cancer has not yet been extensively studied. Studies have demonstrated that PDP2 can inhibit the spread of hepatitis B virus by removing phosphoryl groups from two phosphorylation sites, Ser162 and Thr160, on the core protein [13]. Additionally, PDP2 has been found to regulate lipid metabolism, cell aging, and the differentiation and functional activity of Th17 cells [14–16]. These findings suggest that PDP2 has a range of roles outside of energy metabolism. Consequently, further research is necessary to elucidate the role of PDP2 in breast cancer progression.

Ferroptosis is a type of cell death that is dependent on iron and is characterized by the buildup of lipid peroxides. It is distinct from apoptosis and necrosis since it involves permeability of the cell membrane and is involved in various medical conditions, such as cancer, neurological diseases, and tissue damage[17–21]. This process is initiated by a variety of factors, such as an overabundance of enzymes that generate lipid peroxides, including long-chain acyl-CoA synthetase 4 (ACSL4), lipoxygenase, and NADPH cytochrome P450 reductase, as well as a lack of decomposition, clearance, or reduction capabilities [17–19]. ACSL4 is activated by dimerization and then converts substrate polyunsaturated fatty acids into acyl-CoA [7, 21, 22]. This acyl-CoA is then inserted into the cell’s phospholipid membrane by hemolytic phospholipid transferase 3, which results in the production of lipid peroxides from unsaturated fatty acids and the eventual onset of ferroptosis [23–26].

Phospholipase and phosphokinase are two enzymes that have contrasting effects, thus balancing each other and having opposite functional consequences. Consequently, they should be given equal consideration in research. Our research team, mainly consisting of medical professionals and students of breast surgery, conducted a study utilizing public TCGA data of breast cancer patients to analyze 260 phospholipase genes from the GeneCard database. Through regression analysis, five phospholipase genes were identified as being associated with patient prognosis, with PDP2 exhibiting the highest performance. Additionally, 54 ferroptosis-related genes from the KEGG database were examined, and only 16 showed differential expression. Correlation analysis between these 16 ferroptosis-related genes and the five phospholipase genes indicated that the expression of PDP2 had the strongest correlation with the expression of ACSL4. Subsequently, PDP2 and ACSL4 were selected for further in vitro cell experiments, which demonstrated that PDP2 can inhibit ferroptosis by dephosphorylating ACSL4 in LumABC cells.

## Materials and methods

### Ethics statement

The data used in this study were sourced from public databases, and no animal experiments were conducted in the research, hence negating the need for any related ethical statement.

### Data retrieval and preprocessing

For our research, we retrieved 563 RNA sequencing data of 500 LumABC and 63 adjacent tissues expressed as FPKM values from the Cancer Genome Atlas (TCGA) database of 1,223 BRCA patients, which was downloaded from the UCSC Xena data portal [27]. Furthermore, the clinical data, including survival information and somatic mutation of the LumABC patients, were also acquired from the UCSC Xena data portal. Subsequently, Log2 transformation and quantile normalization was implemented to the RNA-seq data and VarScan2 was employed to analyze the somatic mutation data and compute the tumor mutation burden (TMB) as previously reported [28].

### Analysis of the differentially expressed gene

The "limma" R package was utilized to extract the differentially expressed genes (DEGs) between LumABC and adjacent tissues from 563 RNA sequencing data of 500 LumABC and 63 adjacent tissues. The Benjamini–Hochberg false discovery rate method was then applied to identify genes that were statistically significant and limit false positives [29]. Genes with an adjusted P-value of less than 0.05 and a |log2FC| of at least 1.0 were considered to be statistically significant. The DEGs were visualized by volcano map and heatmap as need.

### Identification of the prognostic phosphatase genes

A total of 260 phosphatase genes were retrieved from GeneCards (https://www.genecards.org/), of which only 28 were identified as DEGs with high expression. These 28 DEGs were further analyzed using a Least Absolute Shrinkage and Selection Operator (LASSO)-penalized Cox regression analysis and a univariate Cox analysis of overall survival (OS) to identify phosphatase genes with prognostic values in the TCGA cohort of LumABC [30, 31]. The results were visualized in a forest plot and a prognostic model, from which five candidate phosphatase genes were selected for further analysis.

### Survival analysis

In order to investigate whether the screening phosphatase or ferroptosis-related genes are associated with the prognosis of patients in the TCGA LumABC cohort, the patients were divided into two groups based on the median risk score values of the corresponding gene expressions. The “Surv” function of the “survival” R package was used to conduct two-sided log-rank tests and Kaplan-Meier survival analyses between the high-risk and low-risk groups, based on the patients’ survival times, in order to determine any differences in overall survival (OS) between the two groups, as reported in a previous study [32].

### Functional enrichment analysis

To gain a better understanding of the potential biological mechanisms between phosphatase PDP2 and ferroptosis, we conducted Kyoto Encyclopedia of Genes and Genomes (KEGG) analysis, gene ontology (GO) analysis, and gene set enrichment analysis (GSEA) based on the DEGs. KEGG is a database that links genomic information with higher-order functional information [33], while GO is a community-based resource that provides information about gene product function through ontologies [34]. The GO analysis was divided into three categories [35]: molecular function (MF), biological process (BP), and cellular component (CC). Furthermore, GSEA was used to explore the pathways and molecular biological functions that PDP2 may affect in ferroptosis [36, 37]. The "clusterProfiler" R package was used to perform functional enrichment analysis, and a statistical threshold criterion with an adjusted P-value < 0.05 was used to identify significant GO terms, KEGG, and GSEA enrichment pathways.

### Analyzing the correlation between the selected phosphatase and ferroptosis-related genes

A total of 55 ferroptosis-related genes (map04216) were retrieved from KEGG database. We choose only the DEGs of the ferroptosis-related genes to analyze their correction with the five screened phosphatases. The DNA expression values of these gene were used to analyze the Pearson correlation between the selected phosphatase and ferroptosis-related genes, which were analyzed and visualized with R package as previously reported [38].

### Western blotting analysis

The sgPDP2 and HA-PDP2 plasmids for gene knockout and overexpression of the PDP2 gene, respectively, were presented by Professor Wei Zhang from Sichuan University for this study. The MCF-7 LumABC cell line was transfected with the sgPDP2 and HA-PDP2, and treated with the ferroptosis inducer erastin (20 μM) in the indicated groups. Cell lysates were then prepared using RIPA buffer and the protein concentration was determined using a Pierce BCA Protein Assay Kit. The lysate was then separated by SDS-PAGE and transferred onto a PVDF membrane. The membrane was blocked with skimmed milk for one hour, followed by incubation with target antibodies (PDP2, ACSL4, pACSL4, or β-actin) overnight at 4°C. Secondary antibodies were then added and incubated for one hour. The membrane was incubated with ECL substrate for 30 minutes and the image was captured using the ECL system.

### Flow cytometry

Flow cytometry was utilized to quantify 7-aminoactinomycin D (7-AAD) positive death cells, lipid peroxidation cells and Fe^2+^, as previously detailed in three distinct studies [39–41]. To measure 7-AAD positive death cells, cells were exposed to 20 μM of the ferroptosis inducer erastin for 24 hours, and then suspended in PBS containing 1 μg/ml of 7-AAD for 10 minutes. These cells were then analyzed by flow cytometry to calculate the percentage of 7-AAD positive cells. Additionally, BODIPY-C11 lipid peroxide was also detected by flow cytometry, as previously reported. Specifically, MCF-7 cells were cultured in triplicate in 12-well plates for 24 hours, followed by treatment with test compounds for the specified duration. Subsequently, cells were incubated with fresh medium containing 2 μM BODIPY 581/591C11 dye (Invitrogen) at 37°C for 20 minutes. After trypsinization, cells were washed with PBS by centrifugation, and the fluorescence intensity of cells with BODIPY 581/591C11 staining was measured by flow cytometry. The fold change of the mean fluorescence intensity (MFI) over the control group, which was shamly treated with normal saline, was then calculated for each sample. To detect Fe^2+^, cells were treated in the same way as 7-AAD and subsequently stained with FerroOrange live cell dye (1 μM, Cat. #SCT210, Merck KGaA, Temecula, CA) at 37°C for 30 minutes. All the flow cytometry analyses were performed using the CyFlow Cube 6 system (Sysmex, Kobe, Japan) and then analyzed and images captured by FlowJo software (BD Biosciences).

### Immunoprecipitation determination

Immunoprecipitation was performed on a whole-cell lysate of MCF-7 cells that had been co-transfected with, or without, sgPDP2 and HA-PDP2 plasmids. The lysate was incubated overnight at 4°C with antibodies against PDP2, ACSL4, and pACSL4, and then incubated for two hours with Protein G Sepharose (Santa Cruz Biotechnology, sc-2002, CA). Subsequently, the immune complexes were washed three times with PBS and analyzed using Western blotting as previously reported [42].

### Transmission electron microscopy

Cells were cultured in a six-well plate and then fixed with 2.5% glutaraldehyde in PBS at 4°C for 4 hours. Following this, the cells were washed three times with PBS for 15 minutes each. Post-fixation was then conducted using 1% OsO4 in PBS for 1.5 hours, followed by three more washes with PBS for 15 minutes. Subsequently, a graded series of ethanol solutions (30%, 50%, 70%, and 80%) was used for dehydration for 15 minutes, followed by a graded series of acetone solutions (90% and 95%) for 15 minutes. This was followed by dehydration in absolute acetone for 20 minutes twice. The cells were then placed in a mixture of absolute acetone and the final Spurr resin in a 1:1 ratio for 1 hour at room temperature and transferred to a 1:3 mixture of absolute acetone and the final resin mixture for 2 hours. Finally, the cells were transferred to a mixture of the final Spurr resin and absolute acetone overnight. After the embedding process was completed, ultrathin sections were cut and stained, and images of the samples were obtained using a transmission electron microscope (H-7650, Hitachi, Japan).

### Statistical analysis

The data were expressed as mean ± SD and statistical analysis was conducted using GraphPad Prism software version 9.0.0 for Windows (GraphPad Software, San Diego, CA). To compare the differences between groups, a one-way ANOVA was used in conjunction with Tukey’s post hoc multiple comparison test, and a significance level of P < 0.05 was established for the adjusted P value.

## Results and discussions

### PDP2 is highly expressed in the LumABC tissues and inversely correlated with patient prognosis

The activities of phosphatases and phosphokinases are in opposition to each other, as phosphatases work to dephosphorylate the phosphate groups that phosphokinases have phosphorylated, thus acting as the "brakes" for phosphokinases. It has been reported by Japanese scholars that PDP2 can directly dephosphorylate the phosphate groups at the phosphorylation sites of Ser162 and Thr160 in the hepatitis B virus core protein [13]. As a group of majorly clinical breast surgeons, we are particularly interested in exploring whether the phosphatases, such as PDP2, secreted by LumABC itself have an effect on the occurrence, progression, and metastasis of breast cancer.

**Fig 1:**
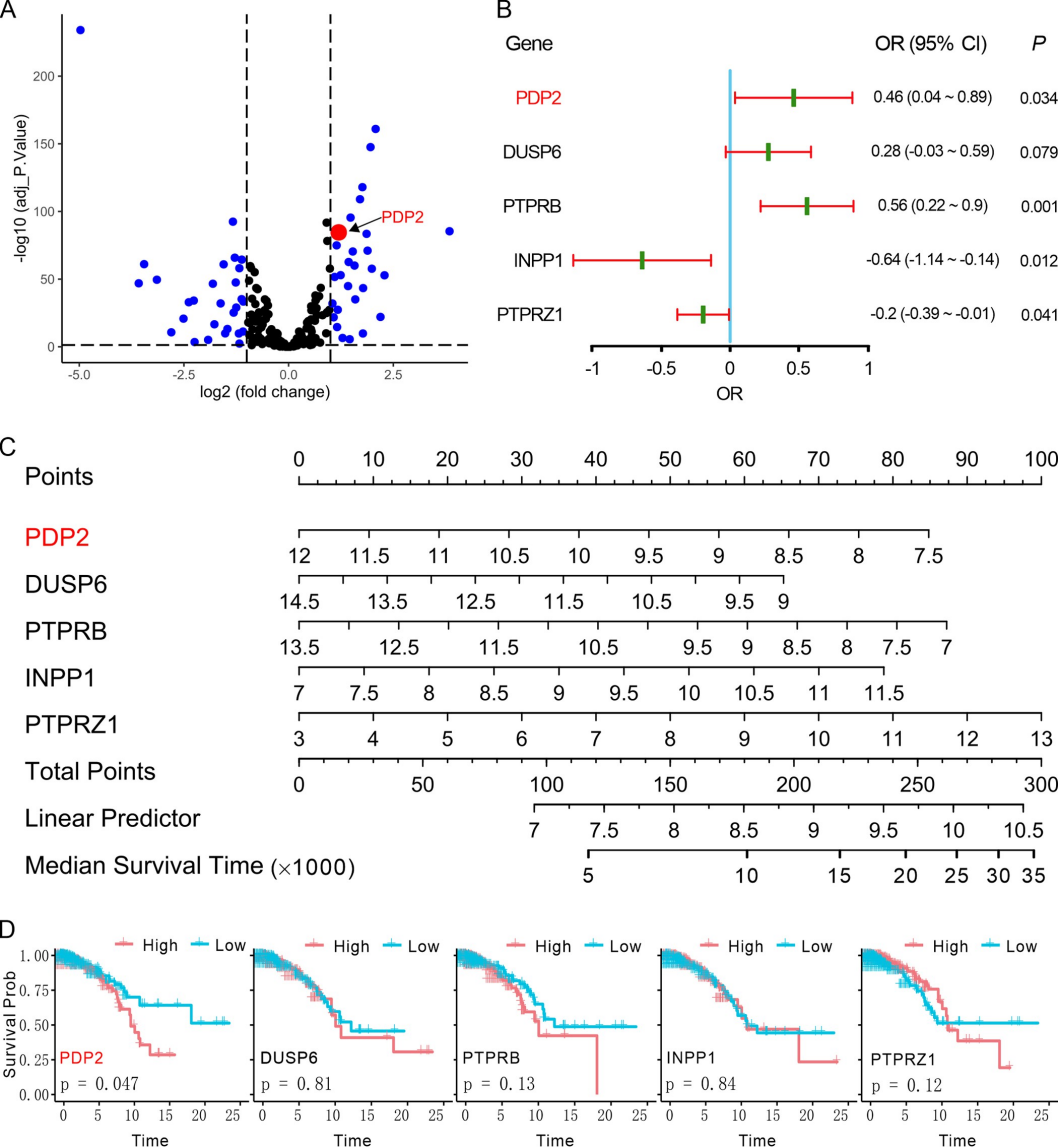
PDP2 is highly expressed in LumABC tissues, and its expression is inversely correlated with patient prognosis. (A) Volcano plot showing the differentially expressed genes (DEGs) between LumABC and adjacent tissues based on data from the TCGA-LumABC cohort. (B) Forest plot showing the LASSO and univariate Cox regression analyses of five phosphatase-related genes based on data from the TCGA-LumABC cohort. (C) Risk diagnostic plot showing the diagnostic values of the five phosphatase genes. (D) Kaplan-Meier survival of the five phosphatases based on gene expression and clinical survival data from the TCGA-LumABC cohort.

To investigate the relationship between the expression of phosphatases and the patient prognosis in LumABC patients, we employed the R package "limma" to analyze the DEGs of 563 RNA sequencing samples, which included 500 LumABC and 63 adjacent tissues, from the TCGA-LumABC cohort. Analysis of the GeneCards database revealed 28 DEGs with high expression (LogFC > 1, adjusted p-value < 0.05). Among these, PDP2 showed the highest expression (Fig 1A). Subsequently, LASSO and COX risk diagnostic models were used to further investigate the 28 genes, and it was found that only the expression levels of PDP2 and four other genes were significantly correlated with the survival of LumABC patients (Fig 1B and 1C). By dividing the LumABC patients into high and low expression groups based on the median gene expression, a significant association between high expression of the PDP2 gene and patient survival time was observed (p = 0.047, Fig 1D). Our bioinformatics analysis suggests that PDP2 is highly expressed in LumABC tissues and that its high expression is inversely correlated with patient prognosis. However, it is also possible that increased expression of PDP2 may not lead to the utilization of the tricarboxylic acid cycle for energy metabolism in LumABC cells, thereby inhibiting the growth and metastasis of LumABC cells. Instead, it may promote the growth and metastasis of LumABC through other unknown mechanisms, resulting in poor prognosis in LumABC patients. Therefore, further research is required to elucidate the underlying mechanisms of PDP2 in LumABC outside the tricarboxylic acid cycle for energy metabolism.

### GO and KEGG analysis indicate abnormalities in multiple pathways related to ferroptosis

GO and KEGG analysis are essential tools for gaining insight into the mechanisms of signaling pathways [43]. By classifying genes and proteins into distinct functional classes based on their molecular functions, biological processes, and cellular components, GO analysis can provide a better understanding of the roles and relationships of different molecules within signaling pathways [44]. Additionally, GO analysis can reveal enriched biological processes, molecular functions, and cellular components associated with a set of genes or proteins [44]. Similarly, KEGG analysis can identify pathways that are significantly enriched with a set of genes or proteins, thereby providing insight into the connections and interactions between various molecules in a signaling pathway [33]. This can aid in the discovery of the underlying mechanisms and regulatory networks, as well as potential drug targets for intervention.

**Fig 2:**
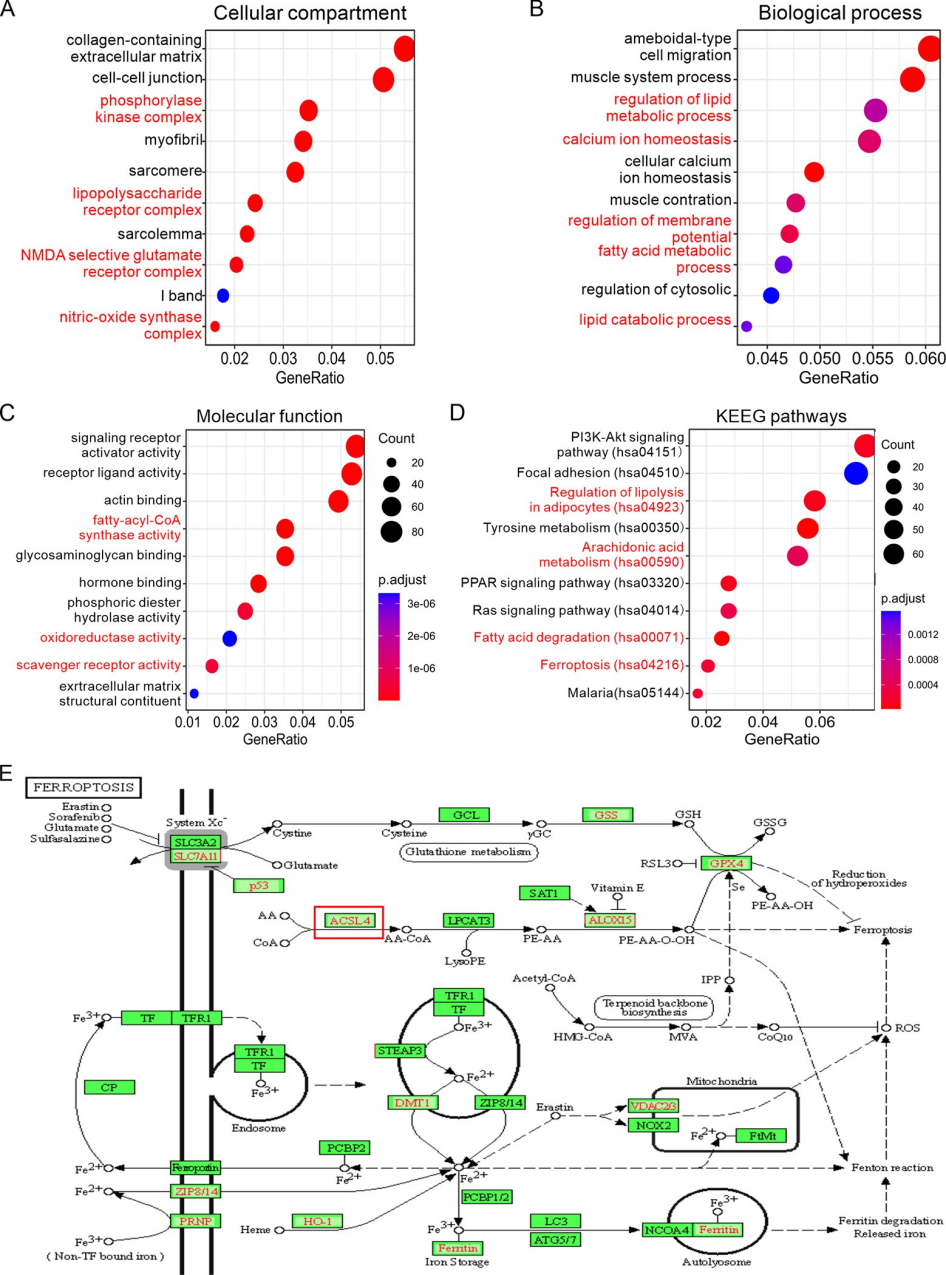
GO and KEGG analysis reveal abnormalities in multiple pathways associated with ferroptosis. (A-C) Results of the GO analysis, showing the first fifteen cellular compartments (A), biological processes (B), and molecular functions (C), respectively. (D) Results of the KEGG pathways analysis, showing the first ten pathways. (E) Visualization of the ferroptosis pathway in the KEGG analysis, showing the connections and interactions among various molecules related to ferroptosis.

To gain a deeper understanding of the effects of PDP2 expression on ferroptosis in LumABC, we conducted GO and KEGG analyses using the DEGs in the TCGA-LumABC cohort. GO analysis revealed that several cellular components, such as the phosphorylase kinase complex, the lipopolysaccharide receptor complex, the NMDA selective glutamate receptor complex, and the nitric-oxide synthase complex (Fig 2A), biological processes, such as the regulation of lipid metabolic processes, fatty acid metabolic processes, and lipid catabolic processes (Fig 2B), and molecular functions, such as fatty-acyl-CoA synthase activity, oxidoreductase activity, and scavenger receptor activity (Fig 2C), were significantly linked to ferroptosis. Consistent with the GO analysis, KEGG analysis revealed that multiple signaling pathways associated with ferroptosis were regulated. Of the first ten most significant pathways, four, namely the regulation of lipolysis in adipocytes, arachidonic acid metabolism, fatty acid degradation, and ferroptosis, were directly linked to ferroptosis (Fig 2D). Additionally, the ferroptosis pathway of the KEGG illustrated multiple mechanisms related to ferroptosis, as indicated by red markers, including ACSL4, which was found to be involved in the DEGs in the TCGA-LumABC cohort (Fig 2E). Taken together, the results of GO and KEGG analysis indicate that high expression of PDP2 may possibly affect the pathways associated with ferroptosis in LumABC.

### GSEA analysis indicates the inhibition of ferroptosis in the TCGA-LumABC cohort

KEGG and Gene Set Enrichment Analysis (GSEA) are two widely used techniques for analyzing signaling pathways in biological research [45]. KEGG is an extensive database that provides information on various biological pathways, such as metabolic pathways, signaling pathways, and diseases [43]. It contains curated pathway maps and gene annotations, allowing researchers to explore the connections between genes and different biological processes. GSEA, on the other hand, is a computational method that evaluates whether a predefined set of genes displays statistically significant differences between two biological states [46]. It does not rely on prior knowledge of specific pathways, but instead focuses on the overall expression pattern of genes within a particular gene set. GSEA ranks all genes according to their correlation with the biological state of interest, and then calculates an enrichment score to determine if the gene set is significantly enriched at the top or bottom of the ranked list [46]. KEGG analysis is beneficial due to its comprehensive pathway database, which provides a great deal of biological knowledge to interpret experimental results [33]. It enables researchers to gain insights into specific pathways and their interactions. GSEA, however, is more versatile and can detect subtle but coordinated changes across multiple genes within a gene set, without being limited by pre-defined pathways [46].

**Fig 3:**
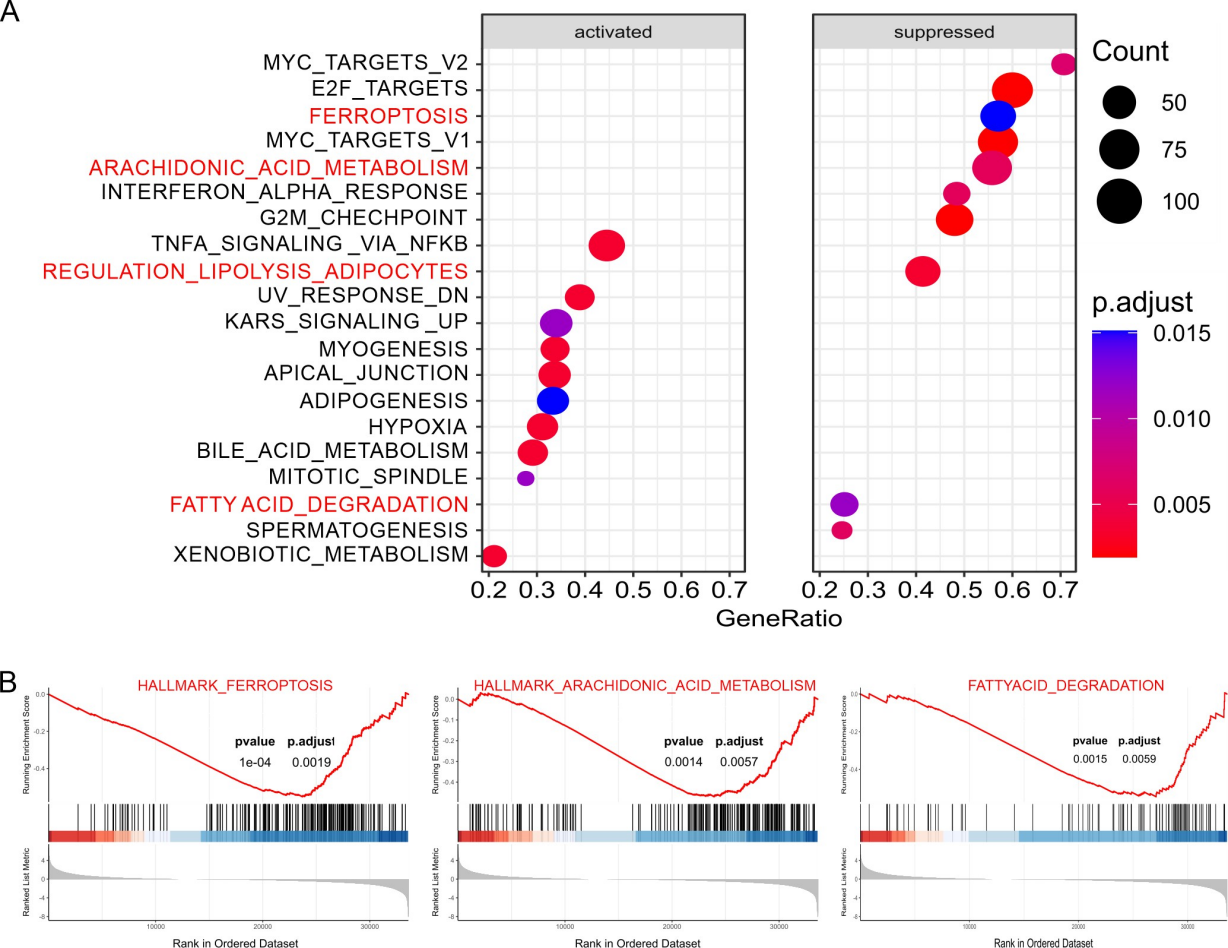
GSEA analysis indicates the inhibition of ferroptosis in the TCGA-LumABC cohort. (A) Thw results of the GSEA analysis show the first twenty pathways. (B) Out of the twenty pathways, three pathways related to ferroptosis are blocked.

In order to evaluate if the DEGs of the TCGA-LumABC cohort present statistically significant differences between LumABC and adjacent tissues, we conducted a GSEA analysis, utilizing the Hallmark gene sets as previously reported [47]. GSEA analysis demonstrated that, of the top twenty most significant pathways, three pathways related to ferroptosis, including ferroptosis, arachidonic acid metabolism, and fatty acid degradation, were suppressed (Fig 3A and 3B). This result was consistent with the KEGG analysis, implying that LumABC cells employed various strategies to impede the production of ferroptosis.

### ACSL4 expression is correlated with PDP2 expression and the prognosis of LumABC patients

Ferroptosis is a type of cell death caused by the lipid peroxidation of cell and organelle membranes, which is mediated by ferrous ions [17–21]. This process is initiated by an excessive production of lipid peroxides, which is due to a lack of decomposition, clearance, or reduction ability. Enzymes such as ACSL4, lipoxygenase, and NADPH cytochrome P450 reductase, which contain iron-containing oxidases, are responsible for generating lipid peroxides [17–19]. Research has suggested that ferroptosis is able to inhibit tumor growth and metastasis; however, tumor cells have developed multiple strategies to evade ferroptosis [23–26]. Thus, it is important to identify the pathways involved in ferroptosis and the mechanisms by which tumor cells evade ferroptosis.

**Fig 4:**
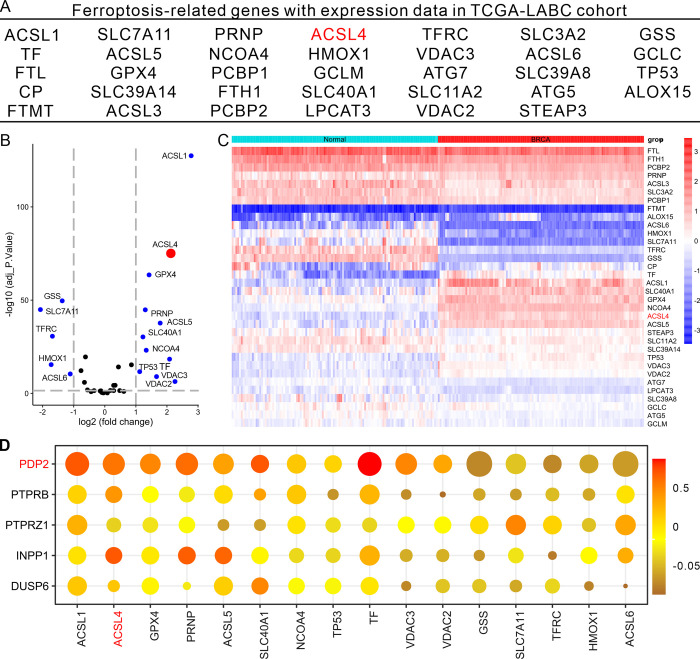
Correlation between ACSL4 and PDP2 expression. (A) ferroptosis-related genes with expression data in the TCGA-LumABC cohort. (B and C) Volcano plots (B) and heatmaps (C) showing differentially expressed genes (DEGs) among the ferroptosis-related genes in the TCGA-LumABC cohort. (D) Correlation analysis of the five phosphatases and the fifteen ferroptosis-related genes.

**Fig 5:**
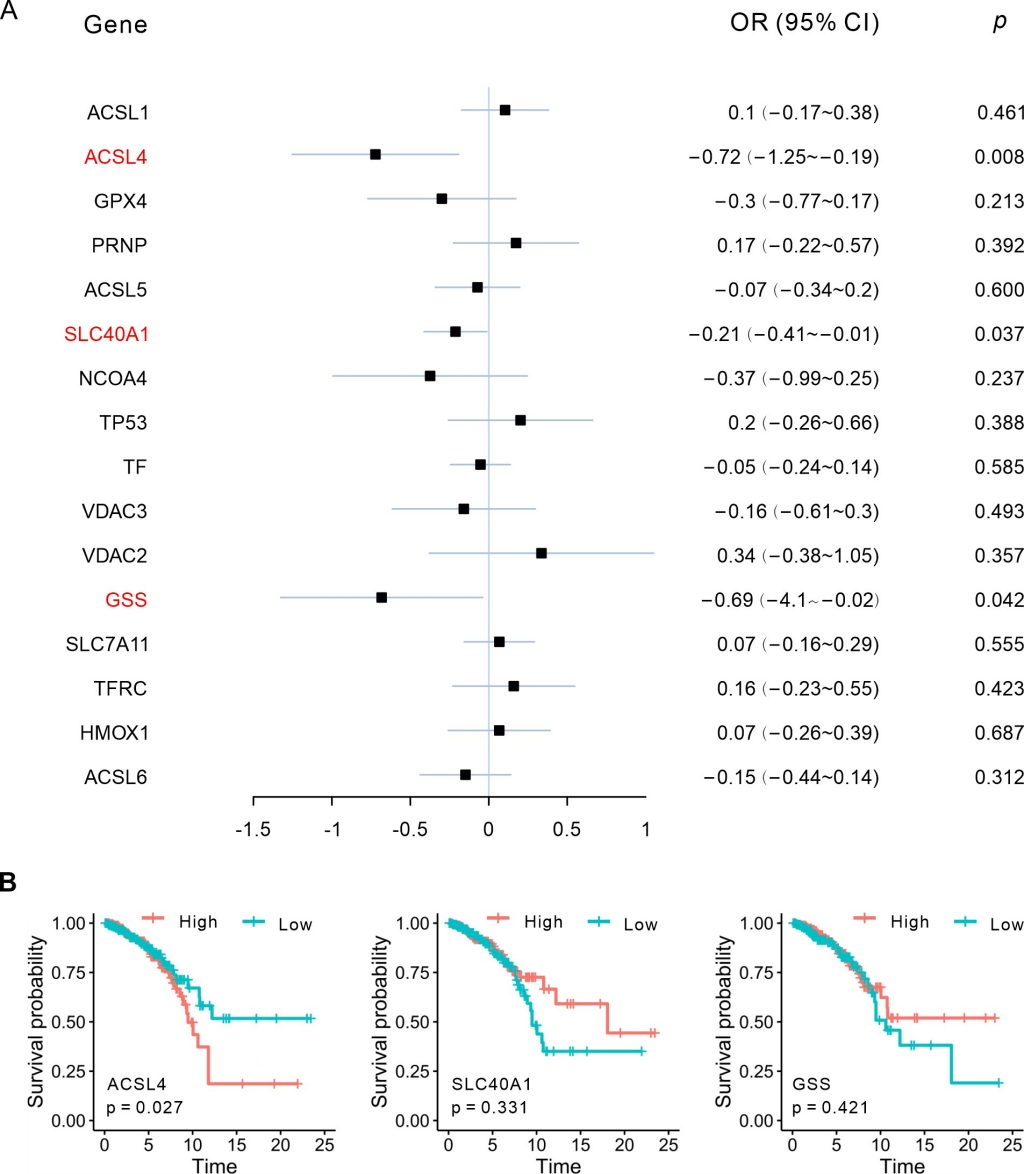
ACSL4 expression is correlated with the prognosis of LumABC patients. (A) Forest plot showing the univariate Cox proportional hazards regression analysis of 16 ferroptosis-related genes based on data from the TCGA-LumABC cohort. (B) Kaplan-Meier survival of the three ferroptosis-related genes based on data from the TCGA-LumABC cohort.

We thus conducted an investigation to identify the correlation between PDP2 gene expression and ferroptosis. We extracted 55 genes from the KEGG database’s ferroptosis pathway, of which 34 had expression data in the TCGA-LumABC cohort (Fig 4A). We therefore examined these genes using volcano plots (Fig 4B) and heatmaps (Fig 4C), and identified ACSL4 and 14 other genes as DEGs, with ACSL4 exhibiting high expression. We then analyzed the correlation between the five phosphatases and the 15 ferroptosis-related genes, and found that the expression of PDP2 had the strongest correlation with the expression of other 15 ferroptosis-related genes, including a significant correlation with the expression of ACSL4 (Fig 4D). We then ran a Cox proportional-hazards analysis on the 15 ferroptosis-related genes and found that only ACSL4, SLC40A1, and GSS had potential diagnostic values associated with the prognosis of LumABC patients (Fig 5A). Thus, to further elucidate the relationship between the expression of these three ferroptosis-related genes and patient prognosis, we divided the LumABC patients into high-expression and low-expression groups based on the median expression level and conducted survival analysis. The results indicated that there was a statistically significant difference in patient prognosis for the ACSL4 (p = 0.027) gene, but not the SLC40A1 (p = 0.331) and GSS (p = 0.421) genes, with higher ACSL4 gene expression being linked to a worse prognosis (Fig 5B).

### PDP2 inhibits ferroptosis through dephosphorylating the phosphorylated ACSL4

The above bioinformatics analysis results indicate that the ACSL4 and PDP2 genes are highly expressed in LumABC and are associated with patient prognosis. Surprisingly, neither of these molecules behaves in the expected manner, as ACSL4 does not promote ferroptosis and PDP2 does not promote the tricarboxylic acid cycle in LumABC cells. This suggests that LumABC cells have adapted the functions of ACSL4 and PDP2 for their own benefit, potentially by overexpressing phosphatase PDP2 and dephosphorylating activated ACSL4 to inhibit lipid peroxidation and ferroptosis, thereby facilitating tumor growth and metastasis.

**Fig 6:**
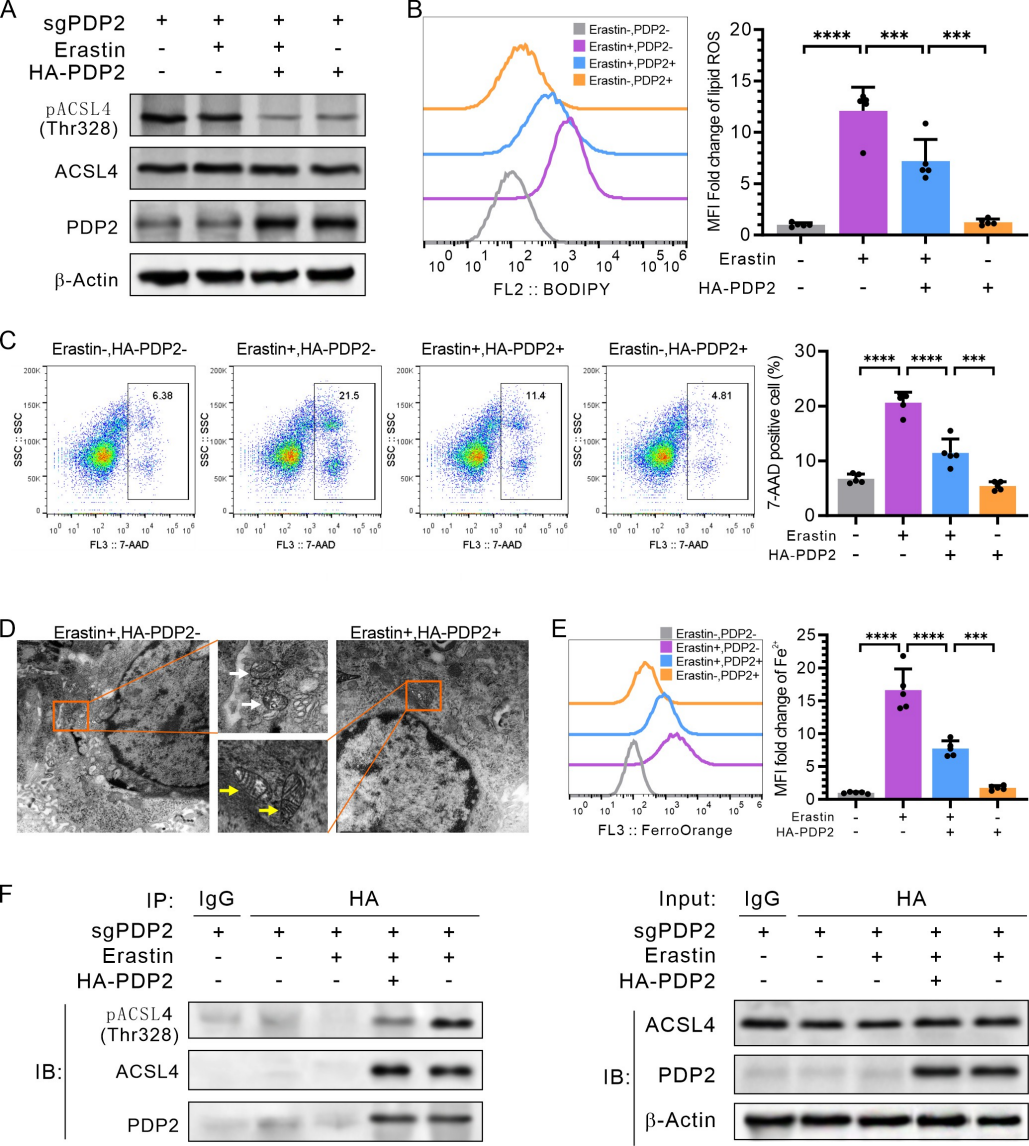
PDP2 inhibits ferroptosis through dephosphorylating phosphorylated ACSL4. MCF-7 cells were treated with the indicated plasmids and erastin. (A) Immunoblotting detection of PDP2, ACSL4, and phosphorylated ACSL4 (pACSL4), showing typical images in three replicates. (B) Flow cytometry detection of lipid peroxides using C11-BODIPY (n = 5). (C) Flow cytometry analysis of the proportion of 7-AAD-positive dead cells (n = 5). (D) Transmission electron microscopy observation of cell morphology, with white arrows indicating ferroptotic mitochondria and yellow arrows indicating normal mitochondria. (E) Flow cytometry detection of the fluorescence intensity of Fe^2+^ in cells stained with FerroOrange (n = 5). (F) Co-immunoprecipitation with anti-HA (PDP2) and immunoblotting detection of PDP2, ACSL4, and pACSL4, respectively. The flow cytometry data represent the mean ± SD from three independent experiments and were analyzed using two-way ANOVA followed by Tukey’s post-hoc multiple comparison analysis. Statistical significance is denoted as *< 0.05, **< 0.01, ***< 0.001, ****< 0.0001.

To validate whether the PDP2 can dephosphorylate activated phosphorylated ACSL4, we conducted the following experiments using the LumABC cell line MCF-7. Since both PDP2 and ACSL4 are expressed in MCF-7 cells, we used a gene editing plasmid, gsPDP2, to knock out the PDP2 gene in MCF-7 cells and simultaneously used a HA-PDP2 expression plasmid for the expression of the PDP2 gene, which was then transfected or not transfected into MCF-7 cells. Upon treatment with the ferroptosis inducer erastin, Western blot analysis revealed a successful suppression of PDP2 expression when the gsPDP2 plasmid alone was transfected into the MCF-7 cells (Fig 6A). Additionally, the level of phosphorylated ACSL4 (pACSL4) was significantly reduced in MCF-7 cells transfected with both gsPDP2 and HA-PDP2 plasmids (Fig 6A).

Ferroptosis is related to iron (Fe^2+^), lipid peroxides, membrane permeability, and specific morphological changes in the nucleus and mitochondria. To determine whether PDP2 can inhibit ferroptosis in MCF-7 cells, membrane permeability dyes 7-AAD and FerroOrange were used to stain the cells. Flow cytometry was then employed to detect the 7-AAD-positive death cells and Fe^2+^ levels. Additionally, lipid peroxide BODIPY was also detected by flow cytometry, and transmission electron microscopy was used to observe the cell morphology. Flow cytometry analysis showed that erastin-treated MCF-7 cells had significantly increased ferroptosis, as indicated by increased production of lipid peroxides BODIPY (p < 0.0001, Fig 6B) and 7-AAD-positive membrane-permeability cell death (p < 0.0001, Fig 6C); however, after co-transfection with gsPDP2 and HA-PDP2 plasmids, both BODIPY staining (Fig 6B) and 7-AAD-positive dead cells (Fig 6C) were significantly reduced (p < 0.001). In addition, transmission electron microscopy of MCF-7 cells treated with gsPDP2 plasmid and erastin revealed an intact nuclear membrane, as well as shrinkage and disappearance of mitochondria with cristae, indicative of ferroptosis morphology. In contrast, cells treated with sgPDP2 plasmid and HA-PDP2 plasmid in combination with erastin displayed a normal morphology (Fig 6D). Flow cytometry results further demonstrated that erastin treatment increased Fe^2+^ ion content compared to cells transfected with sgPDP2 plasmid alone (p < 0.0001), whereas Fe^2+^ ions decreased significantly after treatment with erastin and transfection with HA-PDP2 plasmid (p < 0.0001, Fig 6E). Collectively, these findings suggest that PDP2 expression can effectively inhibit ferroptosis in LumABC MCF-7 cells.

At last, immunoprecipitation experiments revealed that only PDP2 and ACSL4 co-precipitate to form a complex, and only cells with co-precipitated PDP2 and ACSL4 showed a significant decrease in ACSL4 phosphorylation level (Fig 6F). These findings suggest that PDP2 may act as a phosphatase to dephosphorylate and inhibit the activity of ACSL4, which had been phosphorylated and activated.

ACSL4 has been identified as a pivotal component in the process of ferroptosis, a form of regulated cell death characterized by the buildup of lipid peroxides [7, 21, 22]. This enzyme facilitates the incorporation of long-chain polyunsaturated fatty acids (PUFAs) into phospholipids in the cell membrane by catalyzing the esterification of arachidonic acid and other PUFAs with CoA, forming acyl-CoA derivatives [23–26]. These derivatives are then used by enzymes such as lipoxygenases and cyclooxygenases to generate reactive oxygen species (ROS). The accumulation of ROS leads to lipid peroxidation and damage to the cell membrane, ultimately triggering ferroptotic cell death. In this study, our research findings indicated that the expression of PDP2 could diminish the expression of phosphorylated ACSL4, resulting in a substantial decrease in ferroptosis in MCF-7 cells. This was demonstrated by a decrease in lipid peroxide and 7-AAD positive membrane permeability cell death. This is in agreement with our bioinformatics analysis, which suggests that LumABC tumor cells may use the functional activity of PDP2 to dephosphorylate phosphorylated ACSL4 and inhibit the production of ferroptosis. Nevertheless, due to the limited research conducted only in one type of LumABC cell, MCF-7, our evidence is not sufficient to completely confirm the molecular mechanism of PDP2 restraining ferroptosis. Our findings suggest the necessity for further experiments to verify the molecular mechanism of PDP2 inhibiting ferroptosis.

## Conclusion

In this study, we used bioinformatics to analyze the differentially expressed genes (DEGs) between 500 LumABC patients and 63 adjacent tissues in the TCGA-LumABC cohort. Subsequently, we conducted LASSO regression and survival analysis by combining GO, KEGG, and GSEA with the clinical data of the patients. Our results showed that the PDP2 and ACSL4 genes were highly expressed in LumABC tissues and significantly associated with patient prognosis. Furthermore, we knocked out the PDP2 in the LumABC cell line MCF-7. Upon induction of ferroptosis by erastin, we observed that the phosphorylation level of ACSL4 was significantly decreased in the MCF-7 cells transfected with the HA-PDP2 plasmid, and ferroptosis was also significantly reduced compared to the cells without transfection of the HA-PDP2 plasmid. Immunoprecipitation experiments revealed that the phosphorylation level of ACSL4 was only significantly reduced in cells where PDP2 and ACSL4 co-precipitated. These findings suggest that PDP2 may act as a phosphatase to dephosphorylate and inhibit the activity of ACSL4, which had been phosphorylated and activated in LumABC cells. These results also indicate the need for further experiments to confirm the molecular mechanism of PDP2 inhibiting ferroptosis.

## Supporting information

S1 Data (PDF)

S1 File (PDF)
