# Supplementary material for: Autocrine phosphatase PDP2 inhibits ferroptosis by dephosphorylating ACSL4 in the Luminal A Breast Cancer
Source: PLoS One. 2024 Mar 11;19(3):e0299571. doi: 10.1371/journal.pone.0299571 (PMC10927110; doi:10.1371/journal.pone.0299571)

Fig 5A replicate 1 (Cropped in Fig 5A)

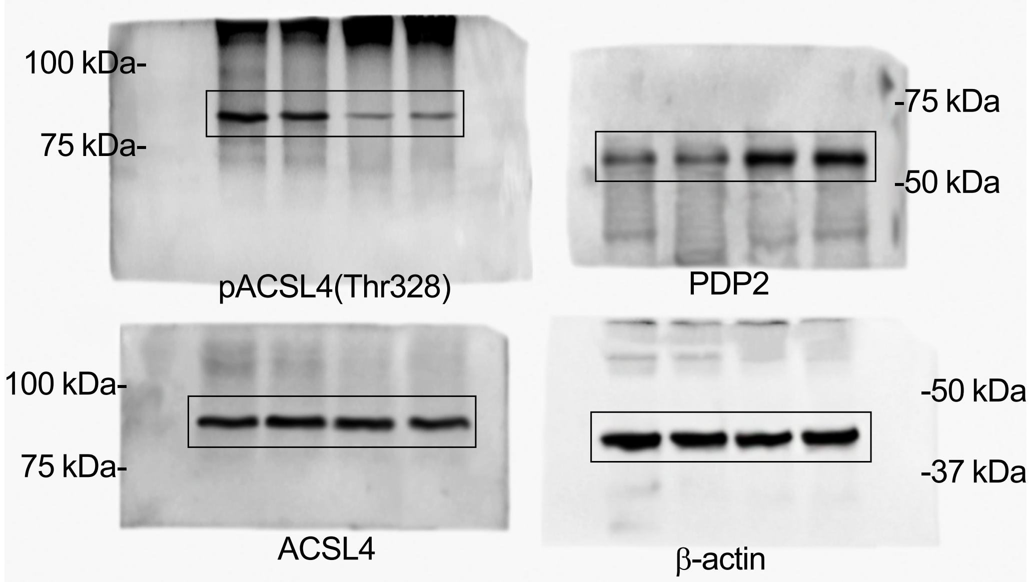

Fig 5A replicate 2

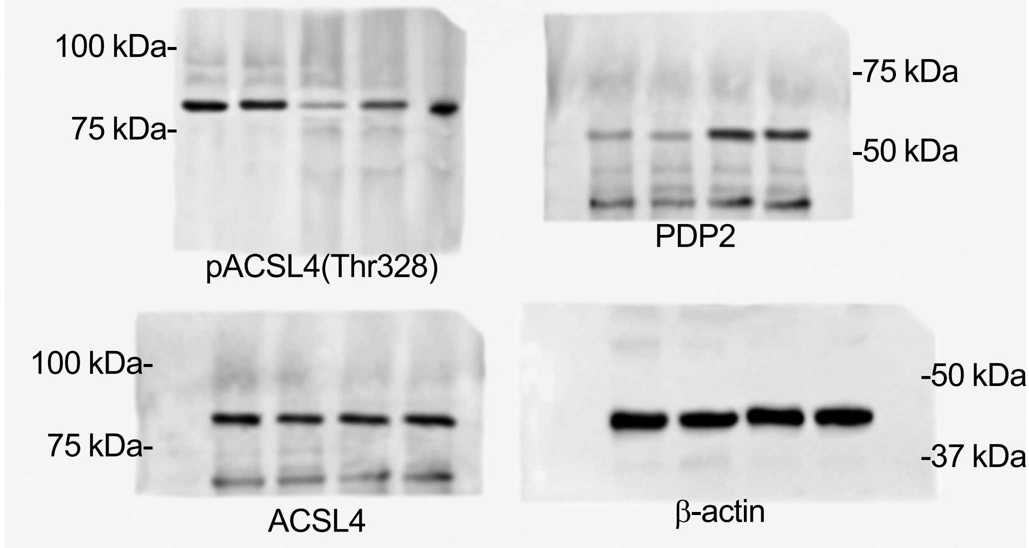

Fig 5A replicate 3

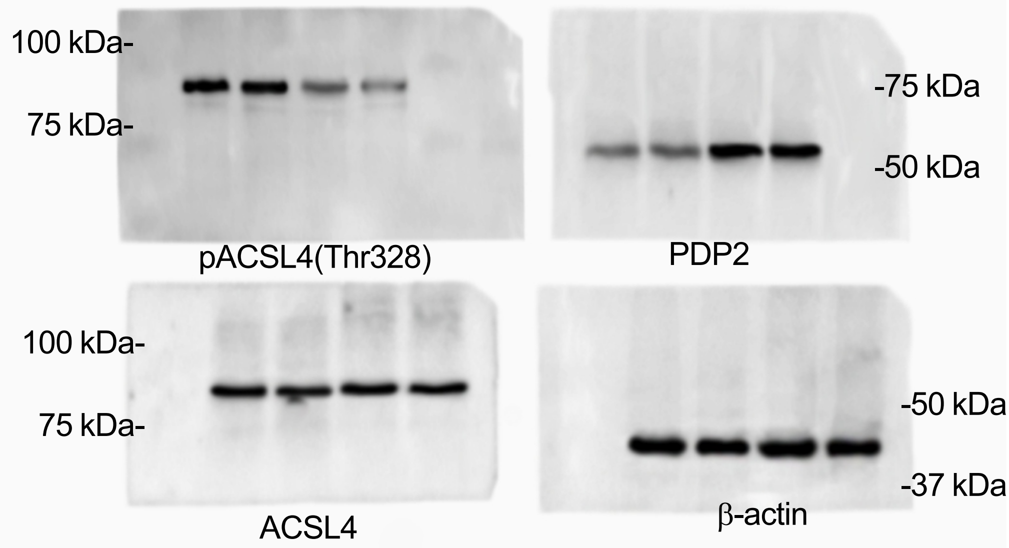

Fig 5F Left panel

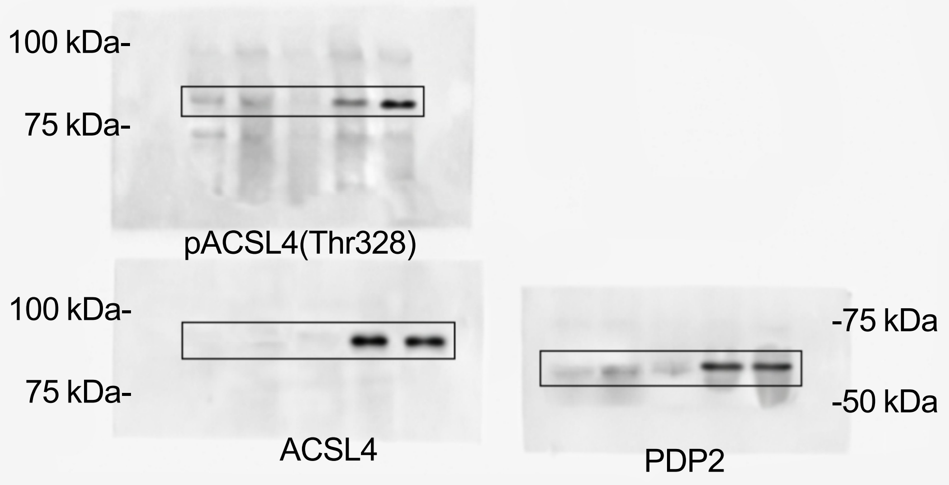

Fig 5F Right panel

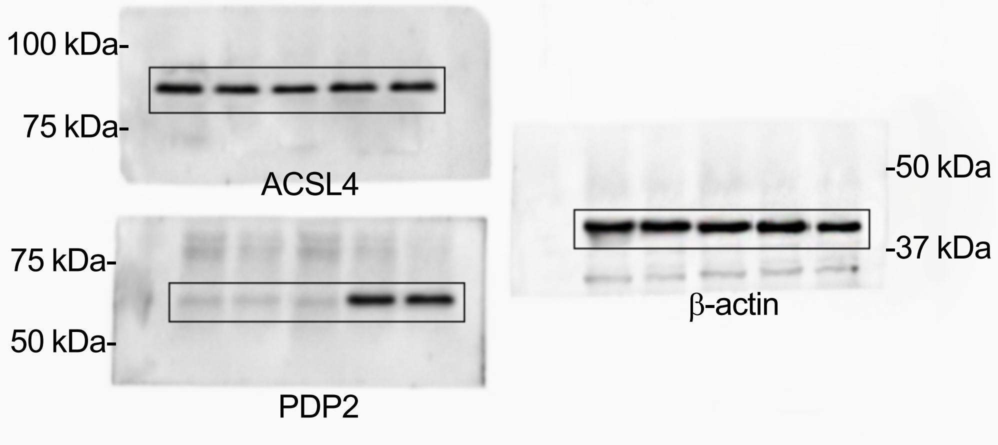

Fig 5B: 5 replicates in each group

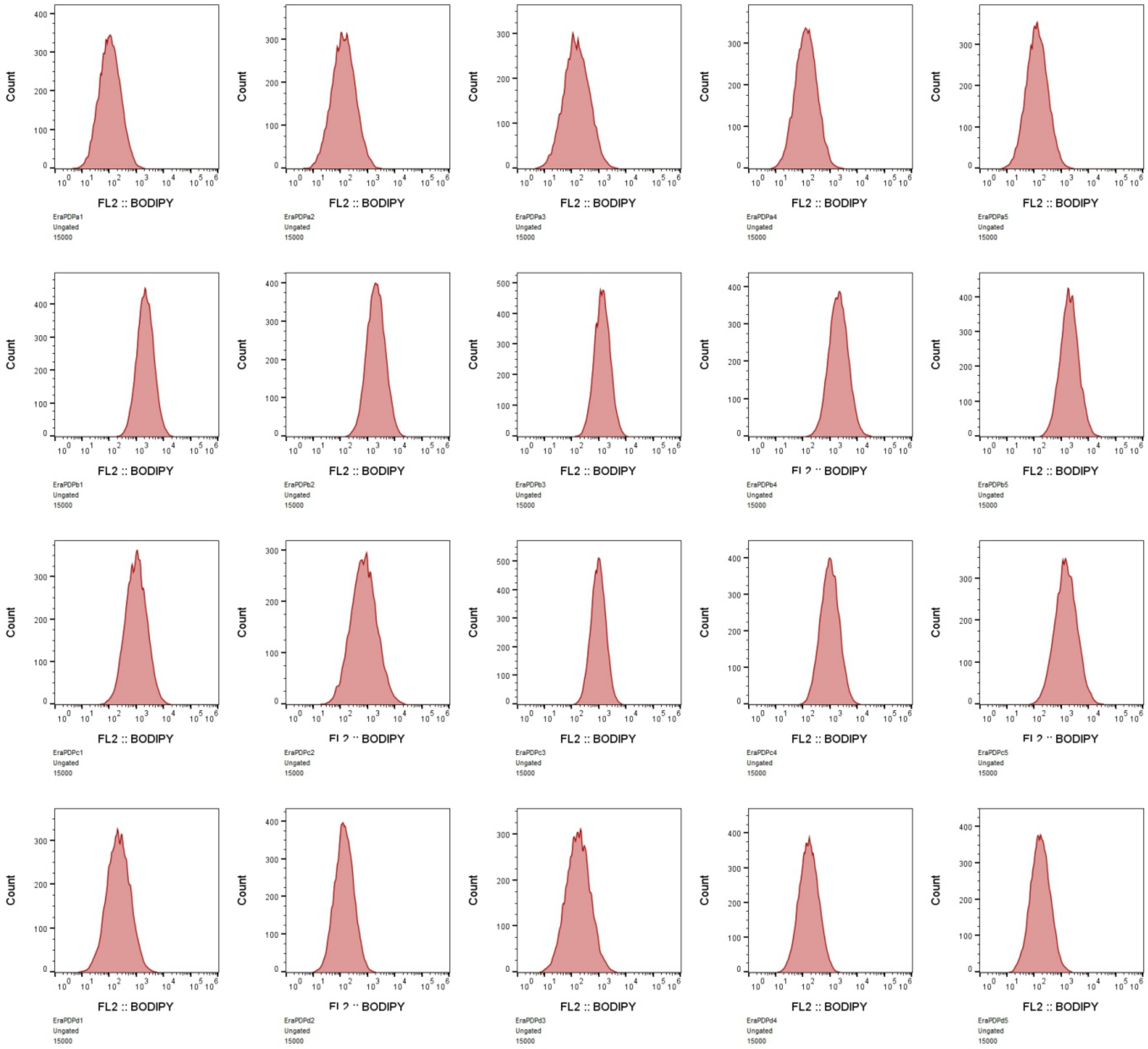

Fig 5C: 5 replicates in each group

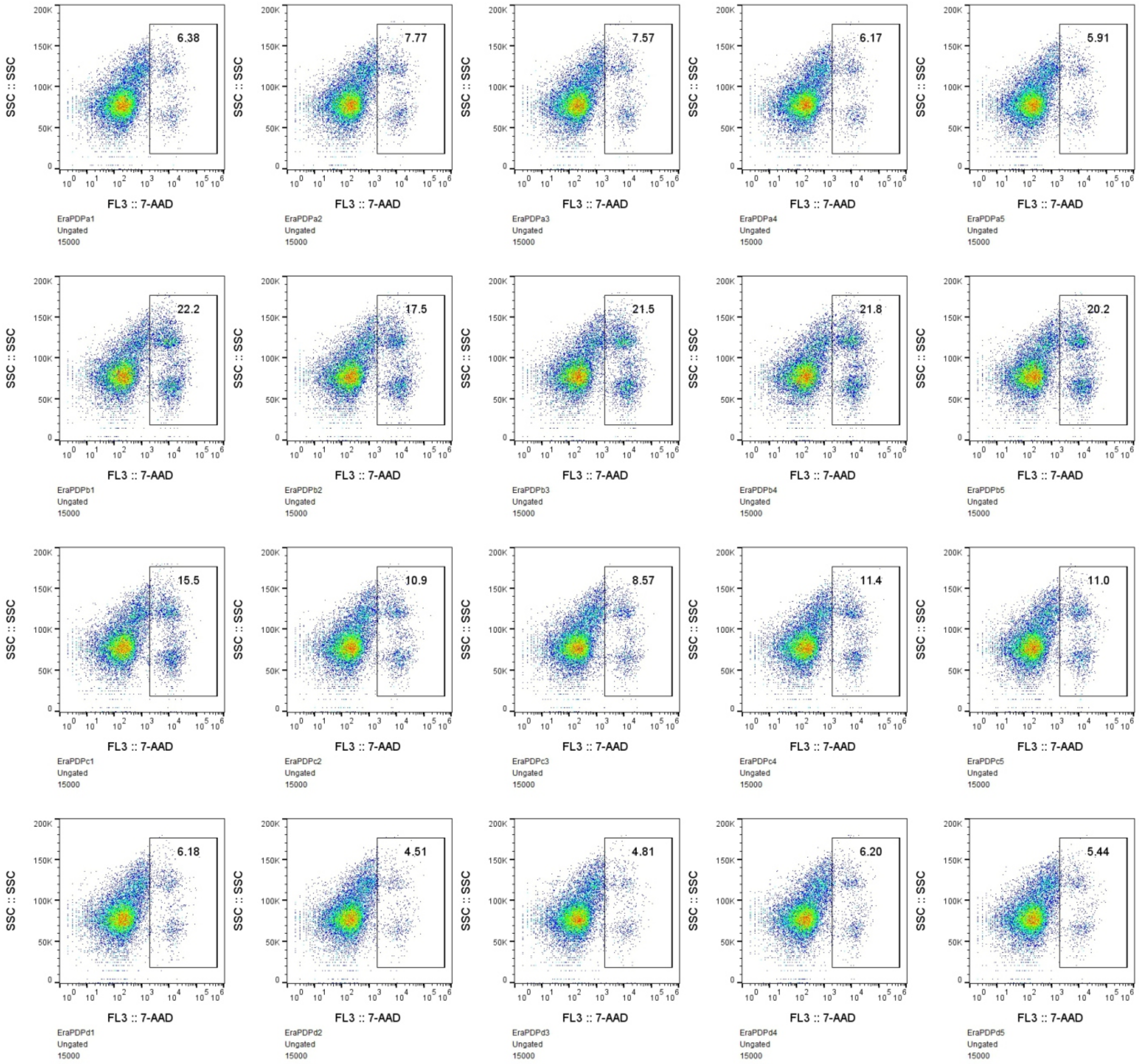

Fig 5B: 5 replicates in each group

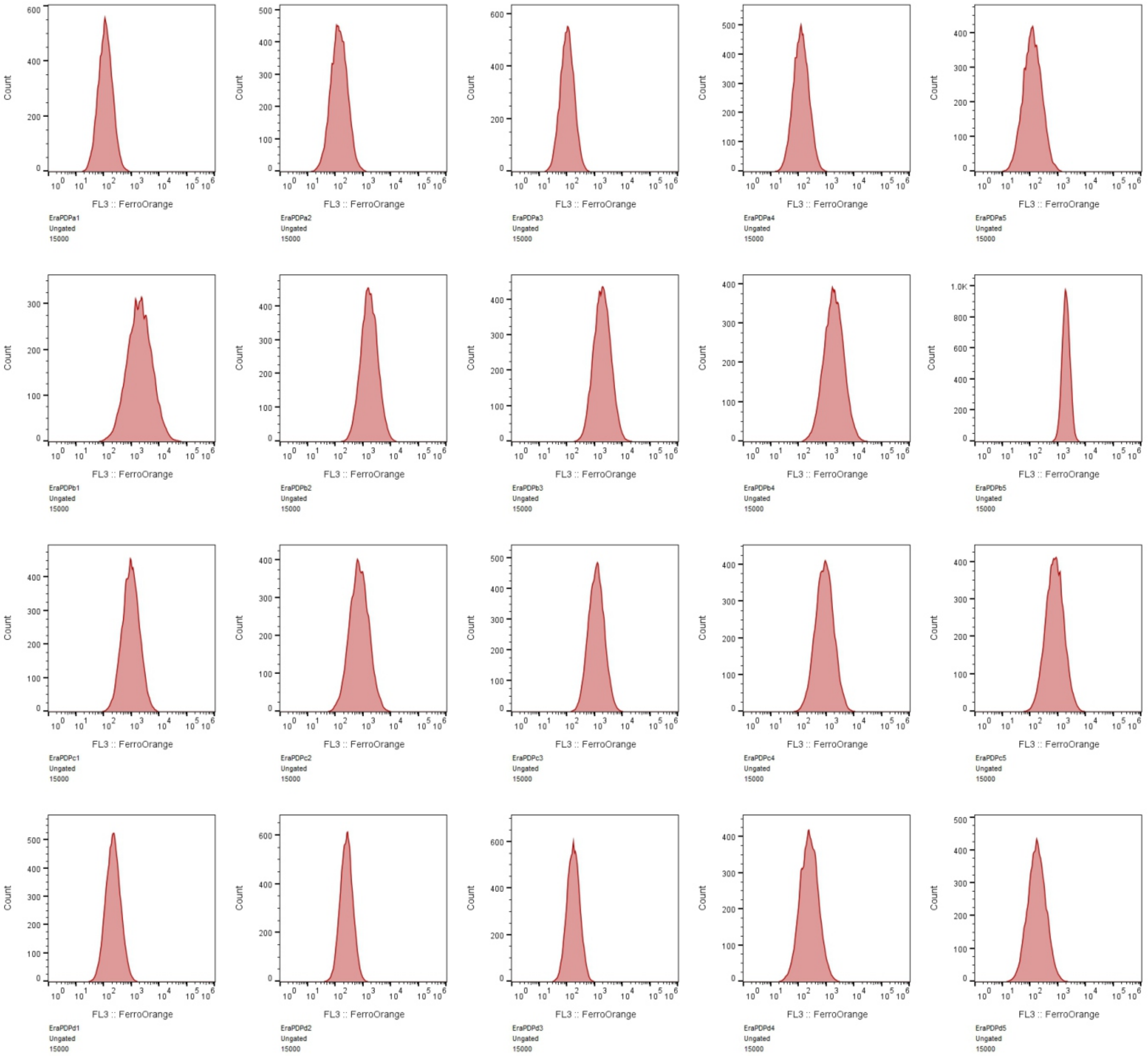

Supplement: S1 Data — (PDF) [file pone.0299571.s001.pdf]
