# Supplementary material for: Autocrine phosphatase PDP2 inhibits ferroptosis by dephosphorylating ACSL4 in the Luminal A Breast Cancer
Source: PLoS One. 2024 Mar 11;19(3):e0299571. doi: 10.1371/journal.pone.0299571 (PMC10927110; doi:10.1371/journal.pone.0299571)
Supplement: S1 File — (PDF) [file pone.0299571.s002.pdf]

R codes for analysis of the relationship between phosphatase and ferroptosis genes

```
################################
#乳腺癌自分泌细胞因子相关分析
#############################################################
#筛选所有乳腺癌来源的个磷酸酶（phosphatase）与发病的关系
library(tidyr)
library(dplyr)
library(tibble)
load(file = "tanAlldif.Rdata")
#load(file = "BRCAclinic.Rdata")
load(file = "tanBRCA_exp-gene_vest.Rdata")
aa1 <- exprSet[grep("ACSL",rownames(exprSet)),]
a <- read.csv(file = "phosphatase.csv")
a1 <- a[grep("Phosphatase",a$Description),] %>% filter(Category=="Protein Coding")%>%
distinct(Gene.Symbol,.keep_all = T)
aa <- a1$Gene.Symbol##这步获得 260 个磷酸酶基因
#write.csv(a1,file = "all_phosphatase_names.csv")
#colnames(BRCAcytokineDiff)
phosphataseDiff <- res %>% filter(res$gene %in% aa) %>%
  arrange(adjPVal) %>% distinct(gene,.keep_all = T) %>% column_to_rownames("gene") %>%
  .[,-1] %>% na.omit() ##这步从 260 个磷酸酶基因中有 256 个基因包含其中，用这个数据作
一个火山图
#####################################################################################
#####################################################################################
###以上 256 磷酸酶个基因的火山图
library(ggplot2)
library(ggrepel)
data <- phosphataseDiff
data$gene <- rownames(data)
ggplot(data=data, aes(x=logFC, y =-log10(adjPVal))) +
  ## 画点
  geom_point(data=subset(data,abs(data$logFC) <= 1),size=2,color="black",alpha=1) +
  geom_point(data=subset(data,data$adjPVal<0.05 & data$logFC >
1),size=2,color="blue",alpha=1) +
  geom_point(data=subset(data,data$adjPVal<0.05 & data$logFC < -
1),size=2,color="blue",alpha=1) +
  geom_point(data=subset(data,data$gene == "PDP2"),aes(size=
abs(logFC)),color="red",alpha=1) +
```

```
    ## 画线
    geom_hline(yintercept = -log10(0.05),lty=5,lwd=0.6,alpha=1)+
    geom_vline(xintercept = c(1,-1),lty=5,lwd=0.6,alpha=1)+
    ## 主题
    theme_bw()+
    theme(panel.border = element_blank(),
              panel.grid.major = element_blank(),
              panel.grid.minor = element_blank(),
              axis.line = element_line(colour = "black"))+
    labs(title="Volcano_plot_by_you", x="log2 (fold change)",y="-log10 (adj_P.Value)")+
    theme(plot.title = element_text(hjust = 0.5))+
    theme(legend.position='none')+
    ## 标签基因名，就是写出基因名
    geom_text_repel(data=subset(data, data$gene == "PDP2"), aes(label=gene),col="black",alpha
= 1)
library(export)
## 导成 PPT 可编辑的格式
graph2eps(file="VocanoPDP2.eps",aspectr=2, font = "Arial",
            height = 5, bg = "transparent")
####################################################################################
########################
difflab <- phosphataseDiff %>% filter(logFC > 1,adjPVal < 0.05)
diffphosphatase <- rownames(difflab)
exprSet <- filter(exprSet,rownames(exprSet) %in% diffphosphatase)
#这里直接筛到 28 个高表达基因，然后下面直接用 COX 多因子分析，找到 5 个 P<0.5 的基因，然后高低分组进行生存分析，确定 PDP2
gc()
############################################################
#筛选复发性乳腺癌来源的个磷酸酶（phosphatase）与发病的关系，这部分也没有用上
library(tidyr)
library(dplyr)
library(tibble)
load(file = "Luminal_A.Rdata")
table(LumAclinic$A2_Event)

load(file = "tanAlldif.Rdata")
#load(file = "BRCAclinic.Rdata")
load(file = "tanBRCA_exp-gene_vest.Rdata")
a <- read.csv(file = "phosphatase.csv")
a1 <- a[grep("Phosphatase",a$Description),] %>% filter(Category=="Protein Coding")
aa <- a1$Gene.Symbol
#colnames(BRCAcytokineDiff)
phosphataseDiff <- res %>% filter(res$gene %in% aa) %>%
    arrange(adjPVal) %>% distinct(gene,.keep_all = T) %>% column_to_rownames("gene") %>%
```

```
    .[,-1] %>% na.omit()

difflab <- phosphataseDiff %>% filter(logFC > 1,adjPVal < 0.05)
diffphosphatase <- rownames(difflab)
exprSet <- filter(exprSet,rownames(exprSet) %in% diffphosphatase)#找出 28 个高表达差异基因用于后面的分析

#save(exprSet,file = "diffphosphatase_exprset.Rdata")
###############################
#https://mp.weixin.qq.com/s/vm3intadGwS5KbX-qaACUw
##############################################
#04 临床预测模型流程化:筛选变量：用 LASSO 方法
library(survival)
library(survminer)
library(rms)
library(ggplot2)
library(glmnet)
gc()
#View(lung)
#整理 cytokine 的表达量的表
load(file = "diffphosphatase_exprset.Rdata") # exprset
exprSet <- data.frame(t(exprSet))
a2 <- which(substring(rownames(exprSet),14,15)=="01")
exprSet <- exprSet[a2,]
dev <- exprSet %>% rownames_to_column("samples")
dev$samples <- substring(dev$samples,1,12)
dev$rowmean <- rowMeans(dev[,-1])
dev <- dev %>% arrange(desc(rowmean)) %>% distinct(samples,.keep_all = T)
dev <- dev[,-30]

a1 <- LumAclinic %>% select(1,4,5)#作 LumA 时
#a1 <- tnbc_sample %>% select(1,4,5)#作 TNBC 时

names(a1) <- c("samples","time","status")
a1 <- filter(a1,a1$time > 1)
dev <- inner_join(a1,dev,by="samples")
dev <- column_to_rownames(dev,"samples")
dev <- na.omit(dev)
dev$status <- ifelse(dev$status=="Alive",1,2)
#save(dev,file = "dev.Rdata")
table(dev$status)
#dev1 <- data.frame(lung)#用这个参考
#for(i in names(dev)[c(5:8)]){dev[,i] <- as.factor(dev[,i])}#本表格标准化了，不变成因子
x = data.matrix(dev[,c(3:30)])
```

```
y = data.matrix(Surv(dev$time,dev$status))

fit = glmnet(x,y,family = "cox")
plot(fit,xvar="lambda",label=TRUE)
cv.fit <- cv.glmnet(x, y, family="cox")
abline(v=log(c(cv.fit$lambda.min,cv.fit$lambda.1se)),lty=2)
plot(cv.fit)
#save(fit,cv.fit,file = "two_fit_lumA.Rdata")#两个文件生成化了一些时间，所以存下来
cv.fit$lambda.min
Coefficients = coef(fit,s=cv.fit$lambda.min)
Active.index = which(Coefficients != 0)
Active.Coefficients = Coefficients[Active.index]
row.names(Coefficients)[Active.index]
#save(a1,Coefficients,Active.index,Active.Coefficients,a2,file = "lumA_lasso.Rdata")
##############################################
##############################################
#从 260 个基因中筛选
#诺莫图；这个比较靠谱：https://blog.csdn.net/anshiquanshu/article/details/53444289
## 第四步 构建模型
library(Hmisc)
library(grid)
library(lattice)
library(Formula)
library(ggplot2)
library(rms)
library(survival)
lung <- dev
#save(dev,file = "dev_lumA.Rdata")
## 添加变量标签以便后续说明
## 第三步 按照 nomogram 要求“打包”数据，绘制 nomogram 的关键步骤,??datadist 查看详细说明。
options(datadist = "dd")

## 构建 COX 比例风险模型，这里用 psm（好像也没有用上），是用后面的 coxph 对 28 个进行缩减的。
colnames(lung)[3:30]
#write.csv(b1,file = "b1.csv")
f2 <- psm(Surv(time,status) ~ DUSP1+PLPP3+PTPN14+PPP1R15A+DUSP6+PPP1R12B+PTPRM+

PPP2R1B+PLPP1+PTPRB+PTPN21+PPM1L+PDP2+ALPL+EEPD1+INPP1+PPP1R16B+EPM2A+

PPP1R1A+PPP1R14A+PLPPR4+PLPP7+PTPRZ1+PPP1R3G+PPP2R2B+PTPRQ+PLPPR1+
            PPP1R17, data = lung, dist='lognormal')
f2 <- psm(Surv(time,status) ~ PDP2+DUSP6+PTPRB+INPP1+PTPRZ1, data = lung, dist='lognormal')
```

```
f2 <- psm(Surv(time,status) ~ PDP2, data = lung, dist='lognormal')
med <- Quantile(f2) # 计算中位生存时间
surv <- Survival(f2) # 构建生存概率函数
## 绘制 COX 回归中位生存时间的 Nomogram 图
nom <- nomogram(f2, fun=function(x) med(lp=x),
                funlabel="Median Survival Time")
plot(nom)

## 绘制 COX 回归生存概率的 Nomogram 图
## 注意 lung 数据的 time 是以”天“为单位
nom <- nomogram(f2, fun=list(function(x) surv(365, x),
                             function(x) surv(730, x)),
                funlabel=c("1-year Survival Probability",
                           "2-year Survival Probability"))
plot(nom, xfrac=0.2)
library(export)
graph2eps(file="预测.eps")
###########################################
##the Cox proportional hazards model
############################################
library("survival")
library("survminer")
library(ggplot2)
## https://www.r-bloggers.com/2016/12/cox-proportional-hazards-model/
res.cox <- coxph(Surv(time, status) ~
DUSP1+PLPP3+PTPN14+PPP1R15A+DUSP6+PPP1R12B+PTPRM+

PPP2R1B+PLPP1+PTPRB+PTPN21+PPM1L+PDP2+ALPL+EEPD1+INPP1+PPP1R16B+EPM2A+

PPP1R1A+PPP1R14A+PLPPR4+PLPP7+PTPRZ1+PPP1R3G+PPP2R2B+PTPRQ+PLPPR1+
                   PPP1R17, data = lung)
res.cox1 <- coxph(Surv(time, status) ~ DUSP6+PTPRB+PDP2+INPP1+PTPRZ1, data = lung)
res.cox2 <- coxph(Surv(time, status) ~ PTPRB+PDP2+INPP1+PTPRZ1, data = lung)
res.cox3 <- coxph(Surv(time, status) ~ PDP2, data = lung)
res.cox
summary(res.cox1)
#作 5 个基因的的森林图
fit.result <- summary(res.cox1)
df1 <- fit.result$coefficients
df2 <- confint(res.cox1)
df3 <- cbind(df1,df2)
df4 <- data.frame(df3[,c(1,5,6,7)])
df4$Var <- rownames(df4)
colnames(df4) <- c("OR","Pvalue","OR_1","OR_2","Var")
```

```
df5 <- df4[,c(5,1,2,3,4)]
df5$OR_mean <- df5$OR
df5$OR <- paste0(round(df5$OR,2), "(", round(df5$OR_1,2), "~", round(df5$OR_2,2),")")
df5$Pvalue <- round(df5$Pvalue,3)

write.csv(df5,file = "forestplot_example.csv",quote = F,row.names = F)
library(forestplot)
fp <- df5[c(3,1,2,4,5),]
forestplot(labeltext = as.matrix(fp[,1:3]),
           mean = fp$OR_mean,
           lower = fp$OR_1,
           upper = fp$OR_2,
           zero = 0,
           boxsize = 0.2,
           graph.pos = 2)

###美化
forestplot(labeltext=as.matrix(fp[,1:3]),
           mean=fp$OR_mean,
           lower=fp$OR_1,
           upper=fp$OR_2,
           zero=0,
           boxsize=0.3,
           lineheight = unit(11,'mm'),
           colgap=unit(4,'mm'),
           lwd.zero=1.5,
           lwd.ci=2,
           col=fpColors(box='#458B00',
                        summary='#8B008B',
                        lines = 'red',
                        zero = '#7AC5CD'),
           xlab="OR",
           lwd.xaxis =1,
           txt_gp = fpTxtGp(ticks = gpar(cex = 0.85),
                            xlab  = gpar(cex = 0.8),
                            cex = 0.8),
           lty.ci = "solid",
           title = "Forestplot",
           line.margin = 0.05,
           graph.pos=2)
####################################################
################################
#用以上 5 个基因作生存曲线分析
library(dplyr)
```

```
gene5 <- gene5 %>%
   # 去掉小于 30 天的
   filter(time >= 30) %>%
   mutate(time = time/365)
### 我们做单个基因的生存分析，这里是单个甲基化位点 cg00018229
rt <- gene5[,c("time","status","PTPRZ1")]
#ifelse 联合 median 快速二分类
rt$status <- ifelse(rt$status == 1,0,1)
rt$risk <- ifelse(rt$PTPRZ1 > median(rt$PTPRZ1),"High","Low")

### logrank 的方法
### 首先 Surv 函数用于创建生存数据对象
library(survival)
surv_object = Surv(rt$time, rt$status)
## 生存数据拟合 survfit
fit1 <- survfit(surv_object ~ risk, data = rt)
summary(fit1)
library(survminer)
ggsurvplot(fit1, data = rt, pval = TRUE)

### 这个里面的是 pval 如何获取呢？survdiff 函数
x = survdiff(surv_object ~ risk, data = rt)
pValue=1-pchisq(x$chisq,df=1)
pValue= round(pValue,3)
pValue
####################################################
####################################################
#用乳腺癌差异基因进行 GO、KEEG 和 GSEA 分析
rm(list = ls())
library(clusterProfiler)
load(file = "diffLab1.Rda")
### 这个分析需要什么数据？？？
### 获得基因列表
library(dplyr)
library(tibble)
difflab <- res %>% subset(logFC >1 & adjPVal < 0.05) %>% .[,-1] %>% na.omit() %>%
   distinct(gene,.keep_all = T)
rownames(difflab) <- difflab$gene
#基因名称转换，返回的是数据框
gene = bitr(gene, fromType="SYMBOL", toType="ENTREZID", OrgDb="org.Hs.eg.db")
#save(gene,file = "gene.Rdata")
#如果网络不好就去掉#号运行下面这个命令，我已经帮大家储存好了
#load(file = "gene.Rdata")
head(gene)
```

```
##################################################
#GO 分析三个大类，细胞组分，cellular compartment
## 这部分需要网络，课堂上不要运行
library(clusterProfiler)
gene <- rownames(difflab)
#基因名称转换，返回的是数据框
gene = bitr(gene, fromType="SYMBOL", toType="ENTREZID", OrgDb="org.Hs.eg.db")
#save(gene,file = "gene.Rdata")
#如果网络不好就去掉#号运行下面这个命令，我已经帮大家储存好了
#load(file = "gene.Rdata")
head(gene)
if(T){
  ego_CC <- enrichGO(gene = gene$ENTREZID,
                     OrgDb= org.Hs.eg.db,
                     ont = "CC",
                     pAdjustMethod = "BH",
                     minGSSize = 1,
                     pvalueCutoff = 0.05,
                     qvalueCutoff = 0.05,
                     readable = TRUE)
}
#save(ego_CC,file="ego_CC_20190316.Rdata")
## 可以加载我存好的数据,
## 但是记住，自己做的时候不能 load 我的数据
#load(file = "ego_CC_20190316.Rdata")
#**作图**
#条带图
barplot(ego_CC)
# 点图
dotplot(ego_CC)

# GO 作图
goplot(ego_CC)
aa <- ego_CC@result
library(export)
## 导成 PPT 可编辑的格式
graph2eps(file="21.eps")
##################################################
#GO 分析三个大类，生物过程 BP,biological process

if(T){
  ego_BP <- enrichGO(gene = gene$ENTREZID,
                     OrgDb= org.Hs.eg.db,
```

```
                                        ont = "BP",
                                        pAdjustMethod = "BH",
                                        minGSSize = 1,
                                        pvalueCutoff = 0.01,
                                        qvalueCutoff = 0.01,
                                        readable = TRUE)
}
#save(ego_BP,file = "ego_BP_20190316.Rdata")
##可以加载我存好的数据
#load(file = "ego_BP_20190316.Rdata")
#**作图**
#条带图
barplot(ego_BP)
#  点图
dotplot(ego_BP)
# GO  作图
goplot(ego_BP)
a2 <- ego_BP@result
###################################################
#GO 分析三个大类，分子功能 MF, Molecular function
if(T){
    ego_MF <- enrichGO(gene = gene$ENTREZID,
                                        OrgDb= org.Hs.eg.db,
                                        ont = "MF",
                                        pAdjustMethod = "BH",
                                        minGSSize = 1,
                                        pvalueCutoff = 0.01,
                                        qvalueCutoff = 0.01,
                                        readable = TRUE)
}
##save(ego_MF,file = "ego_MF_20190316.Rdata")
##  可以加载我存好的数据
#oad(file = "ego_MF_20190316.Rdata")
#**作图**
#条带图
barplot(ego_MF)
#  点图
dotplot(ego_MF)
# GO  作图
goplot(ego_MF)
a3 <- ego_MF@result

#################################################################
#用 KEGG 分析
```

```
##################################################################
library(KEGG.db)
EGG <- enrichKEGG(gene = gene$ENTREZID,
                  organism = 'hsa',
                  pvalueCutoff = 0.05,
                  use_internal_data =T)
##save(EGG,file = "EGG.Rdata")
## 画图
test <- as.data.frame(EGG)
barplot(EGG)
dotplot(EGG)
a4 <- EGG@result
b4 <- a4[grep("PPAR",a4$Description),]
## 可以定制哦！
## rich factor
## 定制一个图片
if(T){
  x = EGG
  ## 内置的函数可以转换为数据框
  df = data.frame(x)
  dd =x@result
  ## 计算富集分数
  dd$richFactor =dd$Count / as.numeric(sub("/\\d+", "", dd$BgRatio))
  ## 提取 p 值小于 0.05 的
  dd <- dd[dd$p.adjust < 0.05,]

  library(ggplot2)
  ## 正式画图
  ggplot(dd,aes(richFactor,forcats::fct_reorder(Description, richFactor))) +
    ## 画横线
    geom_segment(aes(xend=0, yend = Description)) +
    ## 画点
    geom_point(aes(color=p.adjust, size = Count)) +
    ## 调整颜色的区间,begin 越大，整体颜色越明艳
    scale_color_viridis_c(begin = 0.3, end = 1) +
    ## 调整泡泡的大小
    scale_size_continuous(range=c(2, 10)) +
    theme_bw() +
    xlab("Rich factor") +
    ylab(NULL) +
    ggtitle("")
}
graph2eps(file="21.eps")
###########################################################################
```

```
### 使用 clusterProfiler.dplyr 的 mutate 功能
library(clusterProfiler.dplyr)
### richFactor
y=mutate(x, richFactor = Count / as.numeric(sub("/\\d+", "", BgRatio)))
test <- as.data.frame(y)
ggplot(y,showCategory = 30,aes(richFactor,forcats::fct_reorder(Description, richFactor))) +
  ## 画横线
  geom_segment(aes(xend=0, yend = Description)) +
  ## 画点
  geom_point(aes(color=p.adjust, size = Count)) +
  ## 调整颜色的区间,begin 越大，整体颜色越明艳
  scale_color_viridis_c(begin = 0.3, end = 1) +
  ## 调整泡泡的大小
  scale_size_continuous(range=c(2, 10)) +
  theme_bw() +
  xlab("Rich factor") +
  ylab(NULL) +
  ggtitle("")

####################################################################################
######################################
## 还可以看图谱
KEGG_df <- data.frame(EGG)
browseKEGG(EGG, 'hsa04510')

rm(list = ls())
load(file = "EGG.Rdata")
KEGG_df <- as.data.frame(EGG)

options(BioC_mirror="https://mirrors.ustc.edu.cn/bioc/")
if(!require("pathview")) BiocManager::install("pathview",update = F,ask = F)

### 这个分析需要什么啊？？
### 很重要的部分，制作 geneList
### 什么是 geneList
library(clusterProfiler)
load(file = "diffLab.Rda")
gene <- rownames(diffLab)
## 基因名称转换，从 symbol 到 ENTREZID
gene = bitr(gene, fromType="SYMBOL", toType="ENTREZID", OrgDb="org.Hs.eg.db")
## 去重
gene <- dplyr::distinct(gene,SYMBOL,.keep_all=TRUE)
gene_df <- data.frame(logFC=diffLab$logFC,
                      SYMBOL = rownames(diffLab))
```

```
gene_df <- merge(gene_df,gene,by="SYMBOL")

## geneList 三部曲
## 1.获取基因 logFC
geneList <- gene_df$logFC
## 2.命名
names(geneList) = gene_df$ENTREZID
## 3.排序很重要
geneList = sort(geneList, decreasing = TRUE)
## 最好保存一下
## save(geneList,file = "geneList.Rdata")
head(geneList)
library(pathview)
pathway.id = "hsa04110"
pv.out <- pathview(gene.data    = geneList,
                   pathway.id = pathway.id,
                   species       = "hsa",
                   limit           = list(gene=max(abs(geneList)), cpd=1))
## 改变倍数的颜色
pv.out <- pathview(gene.data    = geneList,
                   pathway.id = pathway.id,
                   species       = "hsa"
                   #limit           = list(gene=max(abs(geneList)), cpd=1)
)
## 改变构图
pv.out <- pathview(gene.data    = geneList,
                   pathway.id = pathway.id,
                   species       = "hsa",
                   #limit           = list(gene=max(abs(geneList)), cpd=1),
                   #改变构图
                   kegg.native=F)

###########################################################################
### pathview 批量画图
### 新建文件夹，
### 设置工作目录
### 然后绘图
### 结束后切换回原来的工作目录
dir.create("pathview_out")
## 修改工作目录
if(T){
  for (pathway.id in KEGG_df$ID ){
    pathview(gene.data    = geneList,
             pathway.id = pathway.id,
```

```
                         species        = "hsa"
        )
    }
}

#############################################################
####GSEA 分析来啦，特别有用，一定要掌握!!
##########################################################
## 解释原理
rm(list = ls())
library(clusterProfiler)
load(file = "allDiff.Rda")
#获得基因列表
gene <- res$gene
## 转换
gene = bitr(gene, fromType="SYMBOL", toType="ENTREZID", OrgDb="org.Hs.eg.db")

## 去重
gene <- dplyr::distinct(gene,SYMBOL,.keep_all=TRUE)
#save(gene,file="gene_GSEA.Rdata")
## 网络不好需要加载我保存的 gene
## load(file="gene_GSEA.Rdata")
res1 <- res[res$gene %in% gene$SYMBOL,]
res1 <- distinct(res1,gene,.keep_all = T)
gene_df <- data.frame(logFC=res1$logFC,
                                          SYMBOL = gene$SYMBOL)
gene_df <- merge(gene_df,gene,by="SYMBOL")

## geneList 三部曲
## 1.获取基因 logFC
geneList <- gene_df$logFC
## 2.命名
names(geneList) = gene_df$SYMBOL
## 3.排序很重要
geneList = sort(geneList, decreasing = TRUE)

head(geneList)
library(clusterProfiler)
save(geneList,file = "geneList.Rdata")
###################################################################
####################################################################
### GSEA 变化在于 gene set
####################################################################
### 1.hallmarks gene set
```

```
## 读入 hallmarks gene set，从哪来？
library(clusterProfiler)
load(file = "geneList.Rdata")
hallmarks <- read.gmt("h.all.v7.1.symbols.gmt")
aa <- hallmarks[grep("",hallmarks$term),]
### 主程序 GSEA
y <- GSEA(geneList,TERM2GENE =hallmarks,nPerm = 10000)
yd <- as.data.frame(y)
### 看整体分布
library(ggplot2)
dotplot(y,showCategory=30,split=".sign")+facet_grid(~.sign)
dotplot(y,showCategory=c("HALLMARK_HYPOXIA","HALLMARK_FATTY_ACID_METABOLISM","HALLMARK_GLYCOLYSIS"),split=".sign")+facet_grid(~.sign)+
  scale_y_discrete(labels=function(x) stri_sub(x,10))
### 可以修改标签长度
library(stringi)
library(ggplot2)
dotplot(y,showCategory=10,split=".sign")+
  facet_grid(~.sign)+
  scale_y_discrete(labels=function(x) stri_sub(x,10))

### 选择需要呈现的来作图

library(enrichplot)
gseaplot2(y,"HALLMARK_INTERFERON_ALPHA_RESPONSE",color = "red",pvalue_table = T)
gseaplot2(y,10,color = "red",pvalue_table = T)
gseaplot2(y, geneSetID = 1:3)

### cutting edge 作图
install.packages("ggnewscale")
cnetplot(y,showCategory = 4,foldChange = geneList,colorEdge = T)
cnetplot(y,showCategory = c(1,2,3),foldChange = geneList,colorEdge = T)

library(export)
graph2eps(file="21.eps")
######################################################################
### 筛选特定的通路来画图
### install.packages("clusterProfiler.dplyr-master/",repos=NULL,type="source")
### 代码来自于 Y 叔
### https://yulab-smu.github.io/clusterProfiler-book/chapter13.html
library(clusterProfiler.dplyr)
###
yd <- data.frame(y)
### 提取 NES 大于 0 的，也就是激活的
```

```
y1 <- filter(y,NES > 0)
dotplot(y1,showCategory=30,split=".sign")+facet_grid(~.sign)
y2 <- filter(y,NES < 0)
dotplot(y2,showCategory=30,split=".sign")+facet_grid(~.sign)
### 自定义画图
ggplot(y, showCategory = 30, aes(NES, forcats::fct_reorder(Description, NES))) +
  geom_segment(aes(xend=0, yend = Description)) +
  geom_point(aes(color=p.adjust, size = Count)) +
  scale_color_viridis_c(guide=guide_colorbar(reverse=TRUE)) +
  ##scale_color_continuous(low='red', high='blue', guide=guide_colorbar(reverse=TRUE))+
  scale_size_continuous(range=c(2, 10)) +
  theme_minimal() +
  xlab("Normalized Enrichment Score") +
  ylab(NULL)

####################################################################
### 2.用 KEGG 的通路进行 GSEA 分析
## 读入 kegg gene set
hallmarks <- read.gmt("c2.cp.kegg.v7.1.symbols.gmt")
kegg <- GSEA(geneList,TERM2GENE =hallmarks,nPerm = 10000)
keggd <- data.frame(kegg)
### 看整体分布
dotplot(kegg,showCategory=12,split=".sign")+facet_grid(~.sign)
####################################################################


###############################################################
#下面是分析和铁死亡相关的结果，首先用 TCGA 乳腺癌所有基因，要不行再用亚型，如上面的 LUMNA 或 TNBC
####################################################################################
#####################
library(tidyr)
library(dplyr)
library(pheatmap)
library(tibble)
load(file = "tanAlldif.Rdata")
load(file = "tanBRCA_exp-gene_vest.Rdata")
load(file = "Ferroptosis_names.Rdata")
aa1 <- exprSet[grep("ACSL",rownames(exprSet)),]
aa3 <- data.frame(Ferroptosis_names=rownames(aa1))
Ferroptosis_names <- rbind(Ferroptosis_names,aa3)
Ferroptosis_names <- Ferroptosis_names[-5,]
aa <- Ferroptosis_names
ferroptosis_allDiff <- res %>% filter(res$gene %in% aa) %>%
```

```
  arrange(adjPVal) %>% distinct(gene,.keep_all = T) %>% column_to_rownames("gene") %>%
  .[,-1] %>% na.omit()##这步从 55 个铁死亡基因中有 34 个基因包含其中，用这个数据作一个火山图
b1 <- rownames(ferroptosis_allDiff)
write.csv(b1,file = "ferropname.csv")

#####################################################
###铁死亡火山图
library(ggplot2)
library(ggrepel)
data <- ferroptosis_allDiff
bb1 <- data %>% subset(abs(logFC) > 1 & adjPVal < 0.05)
data$gene <- rownames(data)
ggplot(data=data, aes(x=logFC, y =-log10(adjPVal))) +
  ## 画点
  geom_point(data=subset(data,abs(data$logFC) <= 1),size=2,color="black",alpha=1) +
  geom_point(data=subset(data,data$adjPVal<0.05 & data$logFC > 1),size=2,color="blue",alpha=1) +
  geom_point(data=subset(data,data$adjPVal<0.05 & data$logFC < -1),size=2,color="blue",alpha=1) +
  geom_point(data=subset(data,data$gene == "ACSL4"),aes(size= abs(logFC)),color="red",alpha=1) +
  ## 画线
  geom_hline(yintercept = -log10(0.05),lty=5,lwd=0.6,alpha=1)+
  geom_vline(xintercept = c(1,-1),lty=5,lwd=0.6,alpha=1)+
  ## 主题
  theme_bw()+
  theme(panel.border = element_blank(),
        panel.grid.major = element_blank(),
        panel.grid.minor = element_blank(),
        axis.line = element_line(colour = "black"))+
  labs(title="Volcano_plot_by_you", x="log2 (fold change)",y="-log10 (adj_P.Value)")+
  theme(plot.title = element_text(hjust = 0.5))+
  theme(legend.position='none')+
  ## 标签基因名，就是写出基因名
  geom_text_repel(data=subset(data, data$gene %in% rownames(bb1) ), aes(label=gene),col="black",alpha = 1)
library(export)
graph2eps(file="VocanoPDP2.eps")

##########################################################################
##铁死亡热图
library(tidyr)
library(dplyr)
```

```
library(pheatmap)
load(file = "tanAlldif.Rdata")
load(file = "tanBRCA_exp-gene_vest.Rdata")
load(file = "Ferroptosis_names.Rdata")
#以下找出铁死亡基因,并且是配对的乳腺癌病人的 heatdata
aa1 <- exprSet[grep("ACSL",rownames(exprSet)),]
aa3 <- data.frame(Ferroptosis_names=rownames(aa1))
Ferroptosis_names <- rbind(Ferroptosis_names,aa3)
Ferroptosis_names <- Ferroptosis_names[-5,]
aa <- Ferroptosis_names
heatdata <- exprSet %>% filter(rownames(exprSet) %in% aa)
heatdata <- select(heatdata,-which(substring(colnames(heatdata),14,15)=="06"))
table(substring(colnames(heatdata),1,12))
bb2 <- which(substring(colnames(heatdata),14,15)=="11")
bb1 <- which(substring(colnames(heatdata),14,15)=="01")
normal <- heatdata[,bb2]
#colnames(normal) <- substring(colnames(normal),1,12)
cancer <- heatdata[,bb1]
#colnames(cancer) <- substring(colnames(cancer),1,12)
table(substring(colnames(normal),1,12))
table(substring(colnames(cancer),1,12))
bb1 <- substring(colnames(normal),1,12)
bb2 <- substring(colnames(cancer),1,12)
bb3 <- which(bb2 %in% bb1)
cancer <- cancer[,bb3]
bb4 <- which(bb1 %in% bb2)
normal <- normal[,bb4]
table(substring(colnames(cancer),1,12))
table(substring(colnames(normal),1,12))
which(substring(colnames(cancer),1,12)=="TCGA-A7-A13E")
ab1 <- cancer[,which(substring(colnames(cancer),1,12)=="TCGA-A7-A13E")]
colnames(ab1)
cancer <- select(cancer,-c("TCGA-A7-A13E-01A-11R-A277-07", "TCGA-A7-A13E-01A-11R-A12P-07"))#此前三次才完成删除，所以远行要重复选择三次
#以下才是需足需要的 heatdata
heatdata <- BRCApair
#save(BRCApair,file = "BRCApair.Rdata")
load(file = "BRCApair.Rdata")
table(substring(colnames(heatdata),1,12))
##制作一个分组信息用于注释
group <- c(rep("Normal",112),rep("BRCA",112))
annotation_col <- data.frame(group)
rownames(annotation_col) <- colnames(heatdata)
```

```
#如果注释出界，可以通过调整格子比例和字体修正
pheatmap(heatdata, #热图的数据
         cluster_rows = T,#行聚类
         cluster_cols = T,#列聚类，可以看出样本之间的区分度
         annotation_col = annotation_col, #标注样本分类
         annotation_legend= TRUE, #  显示注释
         show_rownames = T,#  显示行名
         show_colnames = F,#  显示行名
         scale = "column", #以行来标准化，这个功能很不错
         color =colorRampPalette(c("blue", "white","red"))(600),#调色
         cellwidth = 1.5, cellheight = 10,#  格子比例
         fontsize = 5)
##################################################
################################################################################
###
#用磷酸酶 5 个基因和铁死亡 16 个基因进行相关分析
load(file = "BRCApair1.Rdata")#铁死亡 34 个基因

#取出 16 个所谓差异基因的表达值
a <- data.frame(gene16 = rownames(BRCApair1))
##############################
a <-
c("ACSL1","ACSL4","GPX4","PRNP","ACSL5","SLC40A1","NCOA4","TP53","TF","VDAC3","VDAC2","
GSS","SLC7A11","TFRC","HMOX1","ACSL6")
BRCAferro16exp <- BRCApair1[a,]
BRCAferro16exp <- BRCAferro16exp[,113:224]

#取 5 个磷酸酶基因的表达量 C("PDP2","DUSP6","PTPRB","INPP1","PTPRZ1")
load(file = "tanBRCA_exp-gene_vest.Rdata")
b1 <- colnames(BRCAferro16exp)
BRCApha5exp <- exprSet[c("PDP2","DUSP6","PTPRB","INPP1","PTPRZ1"),b1]

m6A_data <- data.frame(t(BRCAferro16exp))
autophagy <- data.frame(t(BRCApha5exp))

#library(dplyr)
library(data.table)
library(ggplot2)
library(reshape2)
library(ggtree)
library(scales)
library(aplot)
#多数据(两组数据的比较)需要使用 ggplot 画气泡图
matrix_MERGE <- data.frame(cor(autophagy,
```

```
m6A_data,method="spearman",use="everything"))#计算相关系数
matrix_MERGE$Autophagy <- rownames(matrix_MERGE)#将多行多列的数字转换为一列数据
data <-
reshape2::melt(matrix_MERGE,id.vars=c("Autophagy"),variable.name="m6A",value.name="r")
##ggplot 作气泡图图：https://mp.weixin.qq.com/s/XyZydfyiy1pBgfwfIgSEHw
  ggplot(data=data,aes(x=m6A,y=Autophagy))+
  geom_point(aes(color=r,size=r*6))+
  scale_color_gradient2(low=muted("red"),mid="yellow",high ="red", midpoint = 0,breaks=seq(-
1,1,0.5))+#颜色渐变
  #scale_color_gradient2(low=muted("blue"),mid="yellow",high =muted("red"), midpoint =
0,breaks=seq(-1,1,0.5))+#颜色渐变
  #scale_color_gradient2(low=muted("blue",l = 10, c = 30),mid="white",high =muted("red",l =
10, c = 30), midpoint = 0,breaks=seq(-1,1,0.5))+#颜色渐变
  scale_size_continuous(range = c(1,8))+#修改点的大小
  guides(size="none")+theme_bw()+
  theme(legend.key.height = unit(3,'mm'),
          legend.justification = c(0,0),
          legend.title = element_blank())+
  theme(axis.text.x = element_text(angle = 90, hjust = 1))#将横坐标标签旋转 90 度
p1
dev.off()
###################################################
#  对铁死亡基因进行 COX 相关分析
##########################################################
gc()
library("survival")
library("survminer")
library(ggplot2)
load(file = "dev_lumA.Rdata")
load(file = "tanBRCA_exp-gene_vest.Rdata")
load(file = "Luminal_A.Rdata")
table(substring(colnames(lung),14,15))
a <-
c("ACSL1","ACSL4","GPX4","PRNP","ACSL5","SLC40A1","NCOA4","TP53","TF","VDAC3","VDAC2","
GSS","SLC7A11","TFRC","HMOX1","ACSL6")
lung <- exprSet[a,]
lung <- lung[,which(substring(colnames(lung),14,15)=="01")]
lung <- data.frame(t(lung))
lung <- rownames_to_column(lung,"id")
lung$id <- substring(lung$id,1,12)
lung <- distinct(lung,id,.keep_all = T)
lung <- dplyr::filter(lung,id %in% rownames(LumAclinic))
lu <- dev %>% select(1:2) %>% rownames_to_column("id") %>% distinct(id,.keep_all = T)
lung <- merge(lu,lung,by="id")
```

```
library(tibble)
lung <- column_to_rownames(lung,"id")

res.cox <- coxph(Surv(time, status) ~ ACSL1+ACSL4+GPX4+PRNP+ACSL5+SLC40A1+NCOA4+
                    TP53+TF+VDAC3+VDAC2+GSS+SLC7A11+TFRC+HMOX1+ACSL6, data =
lung)
res.cox1 <- coxph(Surv(time, status) ~ GPX4+SLC40A1+GSS, data = lung)

res.cox
summary(res.cox1)
summary(res.cox)
summary(res.cox2)
summary(res.cox3)
#作 5 个基因的的森林图
fit.result <- summary(res.cox1)
df1 <- fit.result$coefficients
df2 <- confint(res.cox1)
df3 <- cbind(df1,df2)
df4 <- data.frame(df3[,c(1,5,6,7)])
df4$Var <- rownames(df4)
colnames(df4) <- c("OR","Pvalue","OR_1","OR_2","Var")
df5 <- df4[,c(5,1,2,3,4)]
df5$OR_mean <- df5$OR
df5$OR <- paste0(round(df5$OR,2), "(", round(df5$OR_1,2), "~", round(df5$OR_2,2),")")
df5$Pvalue <- round(df5$Pvalue,3)

write.csv(df5,file = "forestplot_example.csv",quote = F,row.names = F)
library(forestplot)
fp <- df5[c(3,1,2,4,5),]
forestplot(labeltext = as.matrix(fp[,1:3]),
           mean = fp$OR_mean,
           lower = fp$OR_1,
           upper = fp$OR_2,
           zero = 0,
           boxsize = 0.2,
           graph.pos = 2)

###美化
forestplot(labeltext=as.matrix(fp[,1:3]),
           mean=fp$OR_mean,
           lower=fp$OR_1,
           upper=fp$OR_2,
           zero=0,
           boxsize=0.3,
```

```
                lineheight = unit(11,'mm'),
                colgap=unit(4,'mm'),
                lwd.zero=1.5,
                lwd.ci=2,
                col=fpColors(box='#458B00',
                                   summary='#8B008B',
                                   lines = 'red',
                                   zero = '#7AC5CD'),
                xlab="OR",
                lwd.xaxis =1,
                txt_gp = fpTxtGp(ticks = gpar(cex = 0.85),
                                         xlab    = gpar(cex = 0.8),
                                         cex = 0.8),
                lty.ci = "solid",
                title = "Forestplot",
                line.margin = 0.05,
                graph.pos=2)
##################################################################################
# 16 个基因的森林图
################################################################################
fit.result <- summary(res.cox)
df1 <- fit.result$coefficients
df2 <- confint(res.cox)
df3 <- cbind(df1,df2)
df4 <- data.frame(df3[,c(1,5,6,7)])
df4$Var <- rownames(df4)
colnames(df4) <- c("OR","Pvalue","OR_1","OR_2","Var")
df5 <- df4[,c(5,1,2,3,4)]
df5$OR_mean <- df5$OR
df5$OR <- paste0(round(df5$OR,2), "(", round(df5$OR_1,2), "~", round(df5$OR_2,2),")")
df5$Pvalue <- round(df5$Pvalue,3)

write.csv(df5,file = "forestplot_example.csv",quote = F,row.names = F)
library(forestplot)
fp <- df5
forestplot(labeltext = as.matrix(fp[,1:3]),
                mean = fp$OR_mean,
                lower = fp$OR_1,
                upper = fp$OR_2,
                zero = 0,
                boxsize = 0.2,
                graph.pos = 2)

###美化
```

```r
forestplot(labeltext=as.matrix(fp[,1:3]),
           mean=fp$OR_mean,
           lower=fp$OR_1,
           upper=fp$OR_2,
           zero=0,
           boxsize=0.3,
           lineheight = unit(11,'mm'),
           colgap=unit(4,'mm'),
           lwd.zero=1.5,
           lwd.ci=2,
           col=fpColors(box='#458B00',
                        summary='#8B008B',
                        lines = 'red',
                        zero = '#7AC5CD'),
           xlab="OR",
           lwd.xaxis =1,
           txt_gp = fpTxtGp(ticks = gpar(cex = 0.85),
                            xlab  = gpar(cex = 0.8),
                            cex = 0.8),
           lty.ci = "solid",
           title = "Forestplot",
           line.margin = 0.05,
           graph.pos=2)
##################################################
##########################################################################################
################################
#用以上 3 个基因作生存曲线分析
library(dplyr)
gene5 <- lung %>%
  # 去掉小于 30 天的
  filter(time >= 30) %>%
  mutate(time = time/365)
### 我们做单个基因的生存分析，这里是单个甲基化位点 cg00018229
#GPX4+SLC40A1+GSS
rt <- gene5[,c("time","status","GSS")]
#ifelse 联合 median 快速二分类
rt$status <- ifelse(rt$status == 1,0,1)
rt$risk <- ifelse(rt$GSS > median(rt$GSS),"High","Low")

### logrank 的方法
### 首先 Surv 函数用于创建生存数据对象
library(survival)
surv_object = Surv(rt$time, rt$status)
## 生存数据拟合 survfit
```

```
fit1 <- survfit(surv_object ~ risk, data = rt)
summary(fit1)
library(survminer)
ggsurvplot(fit1, data = rt, pval = TRUE)
library(export)
graph2eps(file="s3.eps")
##################################################
##################################################
```
